# Arterial Structural and Functional Characteristics at End of Early Childhood and Beginning of Adulthood: Impact of Body Size Gain during Early, Intermediate, Late and Global Growth

**DOI:** 10.3390/jcdd6030033

**Published:** 2019-09-06

**Authors:** Juan M. Castro, Victoria García-Espinosa, Agustina Zinoveev, Mariana Marin, Cecilia Severi, Pedro Chiesa, Daniel Bia, Yanina Zócalo

**Affiliations:** 1 Departamento de Fisiología, Facultad de Medicina, Centro Universitario de Investigación, Innovación y Diagnóstico Arterial, Universidad de la República, General Flores 2125, 11800 Montevideo, Uruguay; 2 Departamento de Medicina Preventiva y Social, Instituto de Higiene, Facultad de Medicina, Universidad de la República, Alfredo Navarro 3051, 11600 Montevideo, Uruguay; 3 Servicio de Cardiología Pediátrica, Centro Hospitalario Pereira-Rossell, ASSE-Facultad de Medicina, Universidad de la República, Bulevar Artigas 1550, 11600 Montevideo, Uruguay

**Keywords:** adolescents, arterial stiffness, birth weight, body-size trajectories, cardio-metabolic health, children, growth, intima-media thickness, blood pressure

## Abstract

An association between nutritional characteristics in theearlylife stages and the state of the cardiovascular (CV) system in early childhood itself and/or at the beginning of adulthood has been postulated. It is still controversial whether changes in weight, height and/or body mass index (BMI) during childhood or adolescence are independently associated with hemodynamics and/or arterial properties in early childhood and adulthood. Aims: First, to evaluate and compare the strength of association between CVproperties (at 6 and 18 years (y)) and (a) anthropometric data at specific growth stages (e.g., birth, 6 y, 18 y) and (b) anthropometric changes during early (0–2 y), intermediate (0–6 y), late (6–18 y) and global (0–18 y) growth. Second, to determine whether the associations between CVproperties and growth-related body changes depend on size at birth and/or at the time of CVstudy. Third, to analyze the capacity of growth-related body size changes to explain hemodynamic and arterial properties in early childhood and adulthood before and after adjusting for exposure to CV risk factors. Anthropometric, hemodynamic (central, peripheral) and arterial parameters (structural, functional; elastic, transitional and muscular arteries) were assessed in two cohorts (children, *n* = 682; adolescents, *n* = 340). Data wereobtained and analyzed following identical protocols. Results: Body-size changes in infancy (0–2 y) and childhood (0–6 y) showed similar strength of association with CV properties at 6 y. Conversely, 0–6, 6-18 or 0–18 ychanges were not associated with CV parameters at 18 y. The association between CV properties at 6 yand body-size changes during growth showed: equal or greater strength than the observed for body-size at birth, and lower strength compared to that obtained for current z-BMI. Conversely, only z-BMI at 18 y showed associations with CV z-scores at 18 y. Body size at birth showed almost no association with CVproperties at 6 or 18 y. Conclusion: current z-BMI showed the greatest capacity to explain variations in CV properties at 6 and 18 y. Variations in some CV parameters were mainly explained by growth-related anthropometric changes and/or by their interaction with current z-BMI. Body size at birth showed almost no association with arterial properties at 6 or 18 y.

## 1. Introduction

Childhood overweight or obesity have beenshown to be associated with an increase in the prevalence of cardiovascular risk factors (CRFs), cardiovascular (CV) risk and development of CV and metabolic diseases later in life [1,2,3]. This, together with the observed increase in the prevalence of obesity during childhood has led to an increased interest in knowing the factors associated with increased adiposity gain and the link between adiposity gain in early life-stages and future CV alterations; fundamental steps to enable the development of adequate prevention, control and/or treatment strategies. Several studies have been carried out in children and adolescents mainly with the aim of analyzing the association between hemodynamic or arterial characteristics in childhood or adolescence and: (1) body size at birth, (2) growth-related increases in body weight (BW), height (BH), BW-for-BH (BWH), and/or body mass index (BMI), and/or (3) body size at the time of CV evaluation. Results have been heterogeneous. Up to now the factors associated with body size gain as well as the mechanisms underlying the association (impact) of body characteristics and their growth-related changes with CV parameters are not fully known. The controversial findings and lack of a clear understanding of the problem could be attributed to its inherent complexity (e.g., age-related differential impact of body size gain, interaction among co-factors, dependence on prior anthropometric conditions), as well as to characteristics and differences between studies designed to address it (e.g., methodological approaches, publication bias, statistical analyses) [4,5,6].

Early accelerated BW gain (e.g., defined as an increase in BW-for-age z-score of at least 0.67 units between 0 and 2 years old (y)) would be associated with detrimental CV changes in children and adolescents [7,8]. In this regard, at least five interrelated issues should be clarified. First, the strength of the association between CV characteristics (i.e., hemodynamic and arterial) and anthropometric changes (e.g., early BW gain), along with their independence from birth weight and current body conditions.An association between low birth weight (LBW) and CV alterationshas been described, which would be reinforced by (or could even depend on) an accelerated BW gain in the postnatal period and/or by associated excessive subsequent adipose gain expressed by an elevated BMI at the time of CV evaluation [9]. Second, the magnitude of the interactions of anthropometric and CV parameters respect to body size at birth and current BMI should be addressed [10]. Third, the association between BW gain and CV parameters should be analyzed considering that it may depend on the timing of growth-related body changes (e.g., acceleration) [7,8] and/or on thesubject’sage (i.e., at the time of CVassessment) [6,11]. Fourth, as previous works reported that early growthpatterns could be associated with the development or exposure to factors associated with increased CV risk (CRFs’ clustering) [12], the real (independent) clinical meaning or CV impact of BW gain should be determined, taking into account the exposure to CRFs. Fifth, the association between body size gain, birth-size and/or current anthropometric conditions with hemodynamic or arterial parameters could vary, depending on the: (a) arterial type (e.g., muscular vs. elastic); (b) site of evaluation and hemodynamic parameters considered (e.g., peripheral vs. central); and (c) arterial pathway considered (e.g., conductance vs. resistance) [13,14]. Recently, our group demonstrated that z-BMI variations in children and adolescents are positively associated with peripheral (brachial) and central (aortic) blood pressure levels (pBP and cBP, respectively); carotid, femoral and brachial artery diameters; carotid (but not femoral or brachial) intima-media thickness (IMT); arterial stiffness and stiffness gradient. Moreover, we found a “hierarchical order” among hemodynamic and arterial variations associated with z-BMI. pBP was the variable with the greatest variations associated with z-BMI (particularly pBP rather than cBP was associated with z-BMI). The stiffness of central (but not peripheral) arteries was associated with z-BMI (a pBP-dependent association). In turn, variations in arterial diameters were associated with z-BMI, without differences between the elastic and muscular arteries [13]. The above highlights the importance of a comprehensive, multiparametric approach when evaluating the CV system. If results obtained in an arterial segment and/or for a given parameter were generalized, significant errors (biases) could be made.

This work’s aims were: (1) to evaluate and compare the strength of association between hemodynamic and arterial characteristics and (a) anthropometric data at specific growth stages (e.g., birth, 6 y, 18 y) and (b) body size changes during early (e.g., 0–2 y), intermediate (0–6 y), late (6–18 y) and global (0–18 y) growth; (2) to determine whether the association between CV characteristics and growth-related body size changes depends on size at birth and/or at the time of CV evaluation; and (3) to analyze the explanatory capacity of growth-related body size changes for hemodynamic and arterial characteristics in early childhood (6 y) and at the beginning of adulthood (18 y), before and after adjusting for body-size at birth, current BMI and/or exposure to CRFs.

## 2. Materials and Methods

### 2.1. Study Population

The study was carried out in the context of the Centro Universitario de Investigación, Innovación y Diagnóstico Arterial (CUiiDARTE) Project [13,14,15,16]. The protocol was approved by the Institutional Ethics Committee. Both parents’ consent and child’s assent were received before data collection. Participants (or their guardians) signed a written consent prior to the evaluation. Subjects from two cohorts, one of children (*n* = 682) and the other of adolescents (*n* = 340) were included (Table 1). The children cohort, defined based on probabilistic, bi-stage and stratified sampling of subjects attending public kindergartens in Montevideo, is part (subsample) of the longitudinal study “Patrón de crecimiento, estadonutricional y calidad de la alimentaciónen la primerainfancia: análisis de suimpactosobre la estructura y función vascular y el riesgo cardiovascular relativoenniñosuruguayos (CUiiDARTE-Agencia Nacional de Investigación e Innovación(ANII), Ministerio de Desarrollo Social (MIDES), United Nations Children’s Fund(UNICEF) that started in 2010 (first phase) and had in 2016 a second phase [15,16]. In turn, the adolescent cohort (subsample from Montevideo) belongs to a longitudinal (four stages) study called “Estudio Longitudinal del Bienestaren Uruguay” (ELBU) aimed at investigating multidimensional well-being [17] working with a national representative sample of children (and their families) attending in 2004 the first grade of primary public schools in urban areas, which account for 87% of the Uruguayan population.

Similar approaches were carried out on children and adolescents: clinical and anthropometric evaluation, compilation (questionnaires) of data about lifestyle and family history (e.g., educational level, socioeconomic conditions, nutritional factors) and non-invasive CV evaluation.

### 2.2. Anthropometric Evaluation

Anthropometric data (BW and BH) corresponding to ages ≤36 months (mos.) were obtained from registers of the obligatory health-controlsfor children within those ages, established by the Ministry of Health (Children cohort). BW and length at birth in the adolescent cohort were obtained by documented self-report, during parents’ interviews. In turn, following standard procedures, trained technicians obtained anthropometric data from children and adolescents (at participants’ home, school and/or during the CV evaluation). BW and BH were measured with lightweight clothing and without shoes. Standing BH was measured (subject’s head in the Frankfurt Plane position) using a portable stadiometer and recorded to the nearest 0.1 cm. BW was measured with an electronic scale (model 841/843, Seca Inc., Hamburg, Germany; model HBF-514C, Omron Inc., Chicago, Illinois, USA) and recorded to the nearest 0.1 kg. Two measurements were made and a third measurement was obtained in case the first two readings differed by more than 0.5 cm or 0.5 kg. After aggregating records from our technicians and those from health-controls, we obtained BW and BH data corresponding to: (1) birth, 6, 12, 18, 24, 36 and ~72 mos (~6 y) in the children cohort, and (2) birth, ~6 y, ~8 y, ~12 y and ~18 y in the adolescents’ cohort. BMI was calculated as BW-to-squared BH ratio and converted into z-scores (z-BMI). Standardized z-scores for BMI and BWH (up to 2 y), BW-for-age, BH-for-age and BMI-for-age were obtained using World Health Organization software (Anthro-v.3.2.2; Anthro-Plus-v.1.0.4). The changes or differences (Δ) in BWH z-score (ΔBWH z-score) between birth (0 y) and 24 mos. (0–2y, children cohort) were determined. In turn, the changes in z-BMI (ΔBMI z-score) between 0 and 6 y (0–6y, both cohorts), 0 and 18 y (0–18y, adolescents cohort) and 6 and 18 y (6–18y, adolescents cohort) were calculated. Changes were always determined as the difference between the latest (e.g., 18 y) and the earliest (e.g., 0 y) z-score.

### 2.3. Clinical Evaluation

None of the included subjects were taking medications, had congenital, chronic or infectious diseases at the moment of the CV study A brief clinical interview, together with the anthropometric evaluation enabled to assess CRFs exposure. Hypertension, dyslipidemia and diabetes were considered present if they had been previously diagnosed, in agreement with reference guidelines [18]. Subjects <16 y who had brachial systolic and/or diastolic pBP (pSBP and pDBP) >95th percentile for sex, age and BH during the study were considered with high BP levels (HBP); regardless previous diagnosis of hypertension. For subjects aged ≥16 y, HBP levels were defined using cutoff values similar to those for adults (pSBP ≥ 140 mmHg, pDBP ≥ 90 mmHg) [18]. Smokingwas defined as at least one cigarette/week for as long as a month. A family history of CV disease (CVD) was defined by presence of first-degree relatives with premature (<55 y in males; <65 y in females) CVD.

### 2.4. Cardiovascular Evaluation

**Figure 1:**
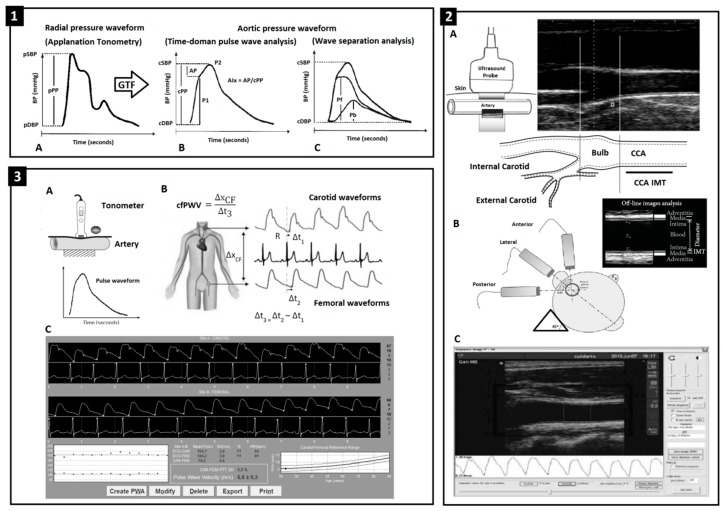
1-A: Radial pulse wave obtained by applanation tonometry (SphygmoCor device); pSBP, pDBP, pPP: peripheral systolic, diastolic and pulse pressure. GTF: general transfer function. 1-B: Aortic wave derived using a GTF; augmented pressure (AP) and augmentation index (AIx) quantified from time-domain pulse wave analysis (PWA). cSBP, cDBP, cPP: central systolic, diastolic and pulsepressure. P1, P2: first and second pressure wave peaks. 1-C: Forward (Pf) and backward (Pb) components’ amplitude obtained from wave separation analysis (WSA). 2-A, 2-B: Methodological approach used to assess common carotid (CCA) and femoral (CFA) artery diameter and intima-media thickness (IMT). Z: acoustic impedance. 2-C: Software for IMT and diameter measurement (Hemodyn-4M). 3-A, 3-B: Methodological approach used to assess carotid-femoral pulse wave velocity (cfPWV). Δx: CCA-to-CFA distance. Δt1, Δt2: time delay between R (ECG) and CCA and CFA foot wave. Δt3: time delay between arterial waves. 3-C: Software for cfPWV measurement (SphygmoCor device).

CV studies were performed in children and adolescents (6 and 18 y, respectively) at the educational centers and/or in CUiiDARTE non-invasive vascular laboratories. The same protocol was applied in both cohorts (Figure 1) and was performed by experienced technicians using the same equipment. In order to reach steady hemodynamic conditions, before starting CV evaluation the subjects hada 10 min rest in a supine position in a quiet, temperature-controlled room.

### 2.5. Peripheral and Central Pressure and Aortic Wave-Derived Parameters

Heart rate (HR), pSBP and pDBP were obtained at 5 min intervals (Hem-4030, OmronInc., Illinois, USA). Peripheral pulse pressure (pPP = pSBP − pDBP) and mean BP (MBP = pDBP + pPP/3) were calculated. To assess cBP and aortic wave-derived parameters, radial artery BP waveforms were recorded using applanation tonometry (SphygmoCor-CvMS, AtCor-Medical, Sidney Australia) (Figure 1). Pressure signals were calibrated topDBP and MBP. A generalized transfer-function (GTF) enabled us to obtain the correspondingcBP waves and central systolic, diastolic and pulse pressure levels (cSBP, cDBP, cPP) [15,19] (Figure 1). Only adequate waveforms (visual inspection) and high-quality recordings (operator index ≥85) were considered. By means of pulse wave analysis (PWA) the first (P1) and second (P2) peaks in cBP wave were identified and their height (amplitude) and time were determined. Then, the difference between P2 and P1 was computed as central augmented pressure (AP) and used to quantify central aortic augmentation index (Aix = AP/cPP). Since AIx depends on HR, AIx adjusted to a 75 beats/minHR (AIx@75) was calculated. Forward and backward (Pf and Pb) components of the aortic pulse wave were also quantified (Figure 1). AIx is a measure of the contribution of reflections to cBP wave amplitude. It depends on the timing and magnitude of the reflected (backward) wave and is influenced by the compliance and structure of vessels distal to the site of measurement, as well as by the distance to the reflection sites. Greater Pb and/or AIx values indicate increased reflections and/or earlier return of reflected waves due to increased arterial stiffness and/or closer reflection sites. Systemic vascular resistance, cardiac output and index were quantified from brachial pulse contour analysis (Mobil-O-Graph, I.E.M.-GmbH, Stolberg, Germany) [15,19]. Only high quality records (index ≤2) and satisfactory waves (visual inspection) were considered. Subjects’ values are the average of at least six consecutive records obtained in a single visit.

### 2.6. Arterial Beat-to-Beat Diameter and Intima-Media Thickness (IMT)

Left and right common carotid and femoral arteries (CCA, CFA) were analyzed using ultrasound (6–13 MHz, M-Turbo, SonoSite Inc., Bothell, WA, USA) and image sequences (30 s, B-Mode, longitudinal views) were stored for off-line analysis. Beat-to-beat diameter waves were obtained using border detection software. Systolic (SD) and end-diastolic (DD) diameters and IMT (far wall, end-diastolic) values were obtained averaging at least 20 beats (Figure 1). CCA diameter and IMT were measured one centimeter proximal to the bulb; CFA diameter and IMT were measured in the straight segment of the penultimate centimeter proximal to the arterial bifurcation (Figure 1) [13].

### 2.7. Local and Regional Arterial Stiffness

CCA and CFA pressure-strain elastic modulus (EM; local stiffness) were calculated as EM = PP/(SD − DD)/DD; cPP and pPP were considered to quantify CCA EM and CFA EM, respectively. Aortic regional stiffness was assessed by means of carotid-femoral pulse wave velocity (cfPWV) (SphygmoCor-CvMS) (Figure 1). The SphygmoCor allowed us to obtain cfPWV from sequential CCA and CFA wave recordings. cfPWV was calculated as the quotient between pulse wave travel distanceand pulse transit time. Real cfPWV was obtained multiplying measured cfPWV by 0.8. cfPWV values were obtained as the mean of three measurements.

### 2.8. Data Analysis and Statistics

A step-wise analysis was performed. First, CV variables were standardized and expressed as z-scores. To this end, subjects not exposed to CRFs (i.e., hypertension, HBP, dyslipidemia, smoking, diabetes, obesity or family history of CVD) were selected from each cohort (reference subgroups: 400 children, 153 adolescents) (Supplementary (S) Table S1). Working with the reference subgroups, mean value (MV) and standard deviation (STD) were determined for each variable (considering age and sex). Then, individual data were converted into z-scores (dimensionless numbers obtained by subtracting the reference MV from an observation, dividing the result by the reference STD) (Table S2) [13].

Second, Pearson product-moment correlations were obtained to quantify the strength of association between CVz-scores and anthropometric variables: (1) at birth: BWH z-score (both cohorts); (2) at the time of the CVstudy: current z-BMI (6 y for children, 18 y for adolescents); (3) growth-related changes: (a) early: ΔBWHz-score 0–2y, (b) intermediate: ΔBMI z-score 0–6y, (c) late: ΔBMI z-score 6–18y and (d) global: ΔBMI z-score 0–18y (Table 2 and Table 3).

Third, statistical comparisons of the correlations’ strengths were done using two-tailed William’s test, making statistical corrections for dependent (same cohort) and overlapping (correlations have a variable in common) variables (e.g., when comparing the correlations “ΔBMI z-score 0–6y and z-pSBP” and “current z-BMI and z-pSBP” in the children cohort) [20] (Table 2 and Table 3). Comparisons between cohorts were made considering William’s test for non-overlapping (no variables in common) and independent (different cohort) variables (e.g., when comparing the R obtained for ΔBMI z-score 0–6y and z-pSBP in children and adolescents) (Table 4).

Fourth, the association between CV z-scores and anthropometric changes during growth was analyzed after statistical adjustment (partial correlations) for: (a) BWH z-score at birth; (b) BWH z-score at birth and current z-BMI and (c) BWH z-score at birth and ΔBMI z-score 6-18y (Table 5, Table 6, Table 7 and Table 8). Multiple linear regression models (MLR; input: enter and forward), enabled to analyze the association between standardized CV data (dependent variables) and (1) single, specific anthropometric changes (ΔBWH z-score 0–2y, Δz-BMI 0–6y, Δz-BMI 0–18y and Δz-BMI 6–18y); (2) BWH z-score at birth; (3) current z-BMI, and (4) the interactions between growth-related changes and birth size or current z-BMI (e.g., (Δz-BMI 0–6y*BWH at birth) and (Δz-BMI 0–6y*current z-BMI)). In other words, since an association between postnatal growth and CV properties might be modified by birth or current body size, interaction between these conditions and growth-related body size changes on CV characteristics was tested adding two product terms (as continuous variables) to the model [21] (Tables S3–S12).

Fifth, using MLR models (input: enter and forward) we analyzed the association between standardized CV variables at 6 and 18 y and anthropometric variables and CRFs (independent variables) (Table 8 and Table 9, Tables S13 and 14). A variance inflation factor (VIF) <5 was selected to evaluate (discard) significant collinearity. 

Analyses were done using MedCalc Statistical Software (v.18.5. MedCalc Inc., Ostend, Belgium); Cocor Statistical Package (http://comparingcorrelations.org/) and IBM-SPSS Software (v.20, IBM-SPSS Inc., Chicago, IL, USA). A *p* ˂ 0.05 was considered statistically significant.

## 3. Results

### 3.1. Arterial System at 6 and 18 y: Comparative Analysis of the Association between Birth or Current Body Size and Early, Intermediate, Late or Global Growth-Related Body Size Changes

The association between CV z-scores at 6 y and (a) body size at birth and 6 y or (b) body size changes during growing-up (0–2 or 0–6y) are shown in Table 2 (children cohort). Both, ΔBWH z-score 0–2y and Δz-BMI 0–6y, were associated with structural and hemodynamic parameters. Positive associations were found between z-pSBP, z-pMBP, z-cSBP, z-cDBP and the ΔBWH z-score 0–2y and Δ z-BMI 0-6y (*p* < 0.05). Negative associations were observed when z-AP, z-AIx and z-AIx@75 were considered. z-Pf was positively associated with Δz-BMI 0-6y (statistical threshold, *p =* 0.053), but the association with ΔBWH z-score 0–2 y did not reach significance (*p =* 0.09). The characteristics and statistical significance of the associations between anthropometric changes and structural properties varied, depending (among on other factors) on the structural parameter considered. No significant associations were found between stiffness z-scores and ΔBWH z-score 0-2 y and Δz-BMI 0–6 y (Table 2)

Unlike BWH at birth, current z-BMI (6y) was associated with hemodynamic and structural parameters. Furthermore, whereas BWH at birth did not show significant associations with hemodynamic variables, current z-BMI was associated (*p <* 0.05) with almost all of them (pBP, cBPandwave-derived parameters) (Table 2).

Compared to ΔBWH z-score 0–2y and Δz-BMI 0–6y, current z-BMI levels (at 6 y) were more strongly associated with CV z-scores. For example, R coefficients for the association between z-pSBP and ΔBWH z-score 0–2y, Δz-BMI 0–6y and current z-BMI (6 y) were 0.16, 0.20 and 0.32, respectively (*p <* 0.05). When compared, the association was stronger in case of z-BMI at 6 y (*p =* 0.019 and *p =* 0.009) (Table 2). The strength of association between arterial parameters at 6 y and ΔBWH z-score 0–2y and Δz-BMI 0–6y did not show statistical differences, with the only exception of z-Right CCA SD, DD and IMT that showed stronger association with 0–6y changes (*p =* 0.026, *p =* 0.002 and *p <* 0.01, respectively).

In adolescents, CVz-scores showed associations with current z-BMI, while almost no association was observed between arterial parameters at 18 y and prior body size characteristics (i.e., BWH at birth and Δz-BMI 0-6y) (Table 3). On the other hand, in case of variables associated with Δz-BMI 0–18y (z-pSBP, z-pPP, z-CCA SD and DD) or BWH z-score at birth (z-Right CCA EM and z-Left CCA EM), the comparative analysis (William’s test) showed that the strongest associations were obtained when considering current z-BMI (18 y) (Table 3).

Jointly analyzing data from both cohorts it was observed that the strength of the associations between CV z-scores and current z-BMI (6 or 18 y), were always greater than those obtained for any change in body size between birth and the time of the study (0–2, 0–6 or 6–18 y) (Table 2 and Table 3).

As mentioned, arterial properties at 6 y were associated with body size changes in that life period (i.e., 0–2 and 0–6 y) whereas the CV properties in subjects 18 y showed almost no association with prior (i.e., Δz-BMI 0–6, 6–18 and 0–18 y) anthropometric conditions (Table 4). When the cohorts were statistically compared, it was observed that for the same “body change” (Δz-BMI 0–6 y), associations were significant for almost all the studied variables when subjects were 6 y, but not when they were 18 y (Table 4). 

Thus, as the subject’s age increases, the association between CV z-scores and prior anthropometric changes (i.e., during childhood) decreases (Table 4).

### 3.2. Arterial Structure and Function at 6 y: Independent Association with ΔBWH z-Score 0–2 y

Associations between hemodynamic and structural parameters at 6 y and ΔBWH z-score 0–2 y kept significant after controlling for BWH z-score at birth (Table 5). After adjusting for BWH z-score at birth and current z-BMI only associations with z-cSBP (but not with z-pSBP, *p =* 0.103) remained significant (*R* = 0.14, *p =* 0.041) (Table 5). Thus, the association between ΔBWH z-score 0-2 y and cSBP, while weak, is independent of size at birth and at the time of CV study. AIx@75 (*p =* 0.009) and Pf (*p =* 0.052) showed significant associations after controlling for body size at birth (Table 5).

As mentioned, significant positive associations were found between ΔBWH z-score 0–2 y and structural parameters. Disregarding BWH z-scores at birth, ΔBWH z-score 0-2y values were positively associated with z-IMT (both CCA) and z-diameters (both CFA, left CCA). Then, the greater the body size change within the first 2 y, the higher the diameters and wall thickness (Table 5). The associations between ΔBWH z-score 0–2 y and z-IMT remained significant after adjusting for BWH at birth and current z-BMI (6 y). Then, regardless of nutritional status at birth and at the time of arterial evaluation, z-IMT levels at 6 y are influenced by ΔBWH z-score 0–2y (Table 5).

There were no significant associations between ΔBWH z-score 0–2 y and stiffness z-scores, before (zero-order correlations) and after (partial correlations) adjusting for BWH z-scores at birth and/or current z-BMI (Table 5).

### 3.3. Arterial Structure and Function at 6 and 18 y: Independent Association with Δz-BMI(Body Mass Index) 0–6 y

Table 6 shows correlations between CV z-scores (at 6 and 18 y) and Δz-BMI 0-6y. In children, z-pSBP, z-pDBP, z-pMBP, z-cSBP, z-cDPB and z-cMBP correlated (positively) with Δz-BMI 0–6 y. Associations remained significant after controlling for BWH at birth, but showed dependence on current z-BMI. AIx and AIx@75 showed significant negative associations when controlling for BWH at birth but when controlling for z-BMI at 6 y, positive correlations were observed (*R*=0.14, *p =* 0.034; *R*=0.19, *p =* 0.005). Both z-CCA IMT were associated with Δz-BMI 0–6 y, with independence of BWH at birth and current z-BMI. Similar results were observed for right and left CFA IMT and DD.

CV parameters (z-scores) at 18 ywere not associated (zero-order correlation) with Δz-BMI 0–6 y. However, several correlations became significant after adjusting for BWH z-scores at birth. Then, body size changes within 0–6 y would contribute to explain CV characteristics at 18 y. Unlike the observed at 6 y, CVparameters at 18 ywere not associated with Δz-BMI 0–6 y (except z-Pb and z-left CCA IMT) after adjusting for body size at birth and current z-BMI (Table 6)

### 3.4. Arterial Structure and Function at 18 y: Independent Association with Δz-BMI 0–18 y and 6–18 y

Table 7 and Table 8 show the association between CV z-scores and Δz-BMI 0–18 and 6–18 y. There were no independent associations between CV properties at 18 y and overall (0–18 y) or late (6–18 y) anthropometric (z-BMI) changes, being the only exceptions z-pPP (for Δz-BMI 0–18 y; *p =* 0.017) and z-Right CCA SD (for Δz-BMI 6–18 y; *p =* 0.035). Then, global changes in body size from birth (0–18 y) or childhood (6–18 y) until late in adolescence would not contribute to explain CV characteristics at the beginning of adulthood, with independence of birth size or current z-BMI.

### 3.5. Hemodynamic and Arterial Properties at 6 and 18 y: Hierarchical Impact of Anthropometric Variables

MLRmodels allowed analyzing whether growth-related body size changes contribute to explain CV properties, considering and comparing BWH z-score at birth, current z-BMI and the interaction of variables (Supplementary Tables S3–S12). In children, cBP and pBP variables (z-SBP, z-DBP, z-PP, z-MBP) were only explained by z-BMI, while wave-derived parameters were explained by z-BMI (z-AIx@75, z-Pf, z-Pb), ΔBWH z-score 0–2 y (z-AIx, z-AP), Δz-BMI 0–6 y (z-AIx) and by Δz-BMI 0–6 y and z-BMI interaction (z-AP). Structural variables were mainly explained by z-BMI; arterial stiffness showed no association with body size parameters (Supplementary Tables S3–S6).

In the adolescents: (1) Δz-BMI 0–6 y showed explanatory capacity when interacting with current z-BMI (both z-CFA IMT) (Supplementary Tables S7 and S8); (2) Δz-BMI 0–18 y showed explanatory capacity when interacting with current z-BMI (z-cDBP, z-Right CFA diameters) or BWH z-score at birth (z-cDBP, z-cPP) (Supplementary Tables S9 and S10) and (3) Δz-BMI 6–18 ydid not showsignificant explanatory capacity for the studied variables (Supplementary Tables S11 and S12). Current z-BMI was always the variable with the greatest explanatory power. The explanatory capacity of the interactions between z-BMI (or z-BWH at birth) and Δz-BMI 0-6, 0–18 or 6–18 y was limited. Compared to current z-BMI, intermediate (0–6 y), late (6–18 y) or global (0–18 y) body-size changes showed almost no explanatory capacityfor interindividual variations in CV properties at 18 y. 

The joint analysis of both cohorts showed that CV variables were mainly associated with current (i.e., 6 or 18 y) z-BMI. Body-size changes showed little individual explanatory power and their contribution was mainly relatedto the interaction with z-BMI at the time of CVevaluation.

### 3.6. Arterial Function at 6 and 18 y: Impact of Body Size Changes vs. Anthropometric and Cardiovascular Risk Factors (CRFs)

Table 9 and Supplementary Table S13 show explanatory models for CV parameters in children. pBP parameters were explained by current z-BMI, while cBP parameters and wave-derivedindexes were mainly associated with z-pSBP. Then, early (0–2 y) or intermediate (0–6 y) body size changes contributed little to explain cBP or pBPvariations found at 6 y compared to the conditions associated with CV risk (i.e., BMI and pSBP) at the time of the CV study.

Explanatory variables for variations in structural parameters showed heterogeneity. At 6 y inter-individual variations in CCA SD and DD were associated with current z-BMI and z-pSBP. When considering CFA z-diameters or z-IMT levels the explanatory variables were: (a) current z-BMI (for z-left CCA IMT, CFA diameter), (b) Δz-BMI 0-6y (for z-Right CFA IMT and diameters), (c) ΔBWH z-score 0–2 y (for z-Right CCA IMT) and (d) BWH z-score at birth (for z-Right CCA IMT). Stiffness variations were not associated with anthropometric variations (Table 9). For the adolescents cohort (Table 10, Supplementary Table S14), the independent variables that remained significant in all the models (*p <* 0.05) were z-pSBP and current z-BMI (Table 10). Then, at 18 y nor BWH z-score at birth, nor the bodily changes during growth (early, intermediate, late or global), explained the CV interindividual variations (with the only exception being the interaction between current z-BMI and Δz-BMI 0-6 y for z-Right CCA IMT) (Table 10).

From the joint analysis of data from both cohorts and considering the exposure to CRFs, it can be asserted that: (1) current z-BMI was the variable mostly associated with CV characteristics; (2) the older the subject, CV properties (e.g., arterial structure) are less explained by changes in body size during the early growth phases.

## 4. Discussion

To our knowledge, this is the first study to describe the association of growth-related changes in body size during early, intermediate, late, or global growth with hemodynamic (central and peripheral) and arterial (structural and functional) properties in early childhood and beginning of adulthood, adjusting for characteristics at birth, at the time of the CV study and for CRFs exposure. Associations were assessed considering three interrelated comparative analysis: (1) strength and (2) independence of the associations and (3) explanatory power of anthropometric parameters and factors associated with CV risk. Our main findings were:

- First, growth-related body size changes (0–2 and 0–6 y) were associated with interindividual variations (z-score) in CV properties at 6 y. Conversely, CV z-scores variables at 18 y were not associated with body size changes (0–6, 6–18 or 0–18 y) (Table 2 and Table 3). Thus, as the subject’s age increases, the association between CV properties and prior body size changes (i.e., during childhood) decreases (Table 4).
- Second, the strength of association between growth-related body changes and CV properties at 6 y was: (a) equal or greater than that observed for body size at birth and (b) lower than the obtained for current z-BMI (at 6 y). Most of the associations between ΔBWH z-score 0–2 y or Δz-BMI 0–6 y and the CV properties at 6 y were independent of the BWH z-score at birth (Table 5 and Table 6). Then, in 6 y children the “hierarchical order” among explanatory variables for CV variations would be: current z-BMI > ΔBWH z-score 0–2 y or Δz-BMI 0–6 y> BWH z-score at birth (Table 2). On the contrary, only current z-BMI showed significant association with CV properties at 18 y (Table 3). In summary, while current z-BMI showed the strongest association, body size at birth showed almost no association with CV properties, regardless of subjects’ age at the time of the CV study (Table 2 and Table 3).
- Third, in general terms, current z-BMI was the anthropometric parameter with the greatest explanatory capacity for CV variations observed at 6 y. Though, variations in some CV parameters were mainly explained by growth-related body changes and/or by their interaction with current z-BMI (Tables S4 and S6). Similar results were observed when the associations were analyzed considering the exposure to CRFs (e.g., hypertension, dyslipidemia) (Table 9). In turn, current z-BMI was the anthropometric variable with the greatest explanatory capacity for CV conditions and variations at 18 y.In summary, body size changes during childhood and/or adolescence contributed to explain arterial variations through the interaction with current z-BMI or BWH z-score at birth (Tables S8, S10 and S12). Among factors associated with CV risk, z-BMI and/or z-pSBP were the main explanatory variables for CV z-scores (Table 10).

The explanatory capacity of growth-related body changes was reduced or lost with, as the age at which the CV system was evaluated increased. In this regard, at least two issues must be analyzed. On the one hand, the impact (association) of anthropometric changes on the CV properties evaluated at a given age, would vary, depending on the period of “body change or gain” considered (e.g., 0–2 y vs. 0–6 y). On the other hand, the association between CV properties and the anthropometric changes observed in a given period (e.g., 0–6 y) could depend on the age at which the CV system is evaluated (e.g., 6 vs. 18 y, like in this work). Studies suggested that BW gain patterns in very early infancy (e.g., 0–6 postnatal mos.) [22,23,24] would be particularly important as determinants of future pBP levels, while other studies showed that BW gain during childhood would be a stronger predictor of pBP [25,26]. The exact “timing of the BW gain” associated with middle or long-term CV risk is still debated, highlighting the need for additional research to clarify and/or reconcile mixed findings. In this work, in general terms we did not find differences in the strength of association when comparing 0–2 vs. 0–6 y data, but some CV characteristics assessed at 6 y showed greater association with 0–6 y anthropometric changes. Evelein et al. (2013) reported that postnatal BW for length gain (0-3 mos.) was associated with carotid IMT (but not stiffness) in children (5 y) [21]. However, when data about growth in later infancy (3–6, 6–9 and 9–12 mos.) were considered, no associations with arterial properties were found [21]. Skilton et al. (2013) reported that BW gain, BH-adjusted-BW gain and ΔBWH z-score 0-18 mos. were positively associated with carotid IMT assessed at 8 y [27]. Unfortunately, the impact of changes in different periods was not analyzed. Linhares et al. (2015) found that the “adverse” long-term effect of accelerated growth in infancy varied depending on the time of growth acceleration. Particularly, carotid IMT at ~30 y was associated with 2–4 y BW-gain, rather than with early BW gain [7]. Additionally, Vianna et al. (2016), found that the relative BW-gain between 2 and 4 y was associated with increased aortic stiffness (evaluated by cfPWV) at ~30 y, whereas birth weight, BW-gain within the first 2 years of life (0–2 y) and linear growth (length/height gain) in childhood were not associated with cfPWV [28]. Pais et al. (2016) assessed PWV in children (8–9 y) and analyzed data considering and categorizing growth trajectories. The highest arterial stiffness levels were observed in groups with accelerated body growth during childhood, with adequate early growth pattern [29].

The dependence on the age at the time of CV study ascribed to the association between an anthropometric parameter and CV properties has been previously described, mainly for birth weight. About this, body size at birth has been associated with pBP levels, andit was reported that the relation becomes progressively stronger with increasing age, being hypothesized that the initiating process occurs in uterus and amplifies throughout life [11,30,31], satisfying theories that seek to explain the detriment of the CV system related to low birth weight and/or catch-up growth [32]. It was even postulated that interactions between increased arterial stiffness, increased pPP, stretching of vascular smooth muscles and synthesis of collagen may contribute to the amplification phenomenon through a feedback loop [33]. By contrast, Lule et al. analyzed data from studies that measured pBP at different ages and did not find an age-related increase in the strength of the association between birth weight and pBP [6]. Furthermore, the relationship between birth weight and later pBP varied depending on the age of the participants: neonates showed consistent positive association; mainly negative associations were seen in children, and studies in adolescents showed inconsistent results [6]. Then, as age increases, the positive association observed in neonates could become negative, non-existent or even positive, which is in agreement with our findings. This could be explained, at least in theory by the fact that as age increases the exposure-time to already present factors capable of impacting on the CV system also increases. As age increases, subjects could become exposed to factors (i.e., CRFs) capable of modifyingCV properties. Then, the association between anthropometricchanges and CVproperties could be modified by exposure to co-factors.

As mentioned in 6 y children the “hierarchical order” among explanatory variables for CV variations would be: current z-BMI >ΔBWH z-score 0–2 y or Δz-BMI 0–6 y> BWH z-score at birth (Table 2). Conversely, only current z-BMI showed significant association with arterial properties at 18 y (Table 3). Birth weight showed almost no association with CV properties, disregard of the subjects’ age at the time of the CV study (Table 2 and Table 3), and most of the associations between ΔBWH z-score 0–2 y or Δz-BMI 0–6 y and CV characteristics at 6 ywere independent of birth conditions (Table 5 and Table 6). Then, the association between body-size changes during infancy or childhood and the CV system at 6 y, would not depend on having been born with low, normal or elevated BWH. When current z-BMI was considered some associations between bodily changes in childhood and CV properties at 6 y were no longer significant. In adolescents, the associations between body changes and CV variables were always dependent on z-BMI at the time of CV study (Table 6, Table 7 and Table 8).

The dependence (or independence) of the association between CV parameters and growth–related anthropometric changes on bodysize at birth and/or on current z-BMI has been previously assessed, with dissimilar findings. A positive association was observed between BW gain or adiposity accumulation during childhood and later pBP levels [23,34,35,36,37]. However, the extent to which birth size modifies the associations between postnatal growth and future pBP levels and/or arterial properties remains unclear. Belfort et al. (2007) found that infants who were thinner at birth were more susceptible to adverse effects on pBP at 3 y of accelerated BWH gain within the first 6 postnatal mos. [22]. Whether the finding is extensive to mid-childhood when BP is highly correlated with adult BP [38] is to be clarified. Leunissen et al. (2012) showed that regardless of birth-size, adiposity accumulation during childhood is a risk factor for later (~20 y) development of high BP levels [37]. Accordingly, Kelishadi’s review (2014) concludes that early growth, rather than birth weight, would be important as a determinant of later BP levels [32]. Supporting a BMI-independent association between body size changes and CV properties, Thiering et al. studied children (*n* = 1127, age≤10 y) and reported that higher BW peak (velocity) in infancy was associated with an increase in pSBP and pDBP after confounders adjustment [39]. In contrast, it has been proposed that the association between BMI at adiposity peak and BP at 6 y would be mediated by current BMI [40]. Marinkovic et al. (2017) observed that infant peak BW velocity and BMI at adiposity peak associationwith childhood pSBP and pDBP (at 6 y), which could be explained by current BMI [12].

Our results support the proposal that the association between anthropometric parameters and pBP depends on current BMI, at the same time as they provide original information showing that unlike what was described for pBP, the association between growth-related body size changes (0–2 y) and cBP at 6 y would not depend on current z-BMI. This is further supported by the fact that reflection parameters, which are main determinants of the differences between cBPand pBP, also showed associations not explained by current BMI (Table 5 and Table 6). It is to note that compared to pBP, the cBP would be of greater value in terms of association with CVchanges and risk prediction [41].

As stated above, the association between growth-related body-size changes and arterial thickness has been previously described [21,27,42]. Our results provide additional information, showing that at 6 y, the association between body size gain and thickness is independent of body size at birth and current z-BMI at 6 y, and that it is statistical significant for both carotid (elastic) and femoral (muscular) arteries. In turn, Evelein et al. (2013) described interaction between birth size and postnatal weight for length by analyzing the impact on arterial stiffness (i.e., distensibility and arterial elastic modulus). The thinner the children were at birth, the lower the distensibility (greater the elastic modulus) with increasing weight for length gain [21]. Then, the impact of birth-size and or growth-related changes would vary depending on the CV properties considered.

Finally, as mentioned, current z-BMI was the anthropometric parameter with the greatest explanatory capacity (power) for the CV variations observed at 6 and 18 y. However, interindividual variations in some hemodynamic and arterial parameters at 6 and 18 y were mainly explained by growth-related body changes and/or by their interaction with current z-BMI (Tables S4 and S6 for children; Tables S8, S10 and S12 for adolescents). Similar results were observed when the associations were analyzed taking into account the exposure to CRFs (Table 9 and Table 10). In children, body change during growth, independently or by means of an interaction with current z-BMI, allowed to explain to a greater extent some CV characteristics (i.e., arterial thicknesses and diameters). In other words, disregard of birth size, exposure to CRFs and/or z-pBP, arterial wall thickness and/or diameters at 6 y could be explained by body growth between 0–2 or 0–6 y. Thus, although CV properties at 6 y would be associated with current z-BMI, knowing the history of BW gain could contribute to a better understanding of the CV characteristics of a specific child. Two children with similar z-BMI, could present CV differences associated with their “history” of body size changes (e.g., between 0–2 or 0–6y). Furthermore, for variables such as wall thickness in children, the history of weight gain would havegreater explanatory capacity than current z-BMI or factors with recognized impact on the CV system (e.g., CRFs). In adolescents, the history of BW gain would not be a primary explanatory variable for CVvariables (i.e., for IMT), but due to variables interactions it could contribute or complement data obtained from current z-BMI and/or BP.

## 5. Strengths and Limitations

This work has several strengths that should be considered. First, the population-based prospective cohort design, including a large number of subjects studied from early life. Repeated measures during growth-period enabled us to study the impact of growth profiles or patterns on CV properties, assessed at two specific times: early childhood (6 y) and onset of adulthood (18 y). Second, we used our own specific “reference populations” to define CV z-scores (Supplementary Tables S1 and S2). Third, many potential confounders were considered in order to isolate the effect of BW gain in the statistical models. Fourth, taking into account that the impact of body change on the CV system may depend on the period in which it occurs, we studied different periods of body gain (0–2, 0–6, 0–18, 6–18 y). Fifth, the relationship between BW gain and adult pBP is one of the most studied, based on the “fetal origin” hypothesis, but pBP is a particular variable and does not inform about central hemodynamic conditions, or about structural and/or functional arterial changes (e.g., associated with early vascular aging or atherosclerosis development). Thus, we designed an integral approach in which multiple CV parameters (e.g., pBP, cBP, arterial diametersand thicknesses, local and regional stiffness) and different arterial pathways (i.e., elastic and muscular) were evaluated. Sixth, unlike most works that analyzed the associations between body changes and the CV system considering a single age, we studied children and young adults. Up to now, most studies included premature, small for gestational age, obese and/or hypertensive subjects and data about the CVimpact of growth-related bodychanges in healthy pediatric and/or adolescent populations werescarce. In this work, healthy children and adolescents were studied.

Some limitations should be considered. First, we did not have information about blood biomarkers measured by our technicians. Therefore data about some conditions (i.e., existence of dyslipidemia) was obtained from reference physicians, registers and/or self-reports. Second, although we adjusted for several potential confounders, residual confounding factors may persist, as in any observational study. Third, in this work we chose to use change in BWH z-score or z-BMI between two time points as growth-indicators. This approach is a simple practical (clinical) method for quantifying a “change”; although more detailed growth patterns could be derived from longitudinally collected anthropometric measures in both cohorts. Fourth, we did not perform an analysis discriminating by sex; despite we are aware of data suggesting that the impact of childhood growth on the CV system may differ between boys and girls [12]. Fifth, we included subjects born at term and preterm, but as most of them belonged to the first condition (98% and 92% inchildren and adolescent cohorts, respectively) the results should be assigned to term-born subjects. Sixth, comparative analysis of the associations between anthropometric data and CV (hemodynamic and/or arterial) variables measured at 6 and 18 y was done considering two different cohorts, instead of a single cohort followed for more than 20 years. Although obtaining similar data for different cohorts could be considered as strength of the work, as a limiting factor it should be noted that for some variables data were not obtained in both cohorts and some aspects of the associations could only be evaluated in one of them. Finally, we did not analyze growth considering body composition (e.g., fat mass) and its changes as was previously done [43].

## 6. Conclusions

Body-size changes in infancy (0–2 y) and childhood (0–6 y) showed similar strength of association with respect to CV properties assessed at 6 y. Conversely, changes between 0–6, 6-18 or 0–18 y were not associated with CV parameters evaluated at 18 y.

The association between CV characteristics at 6 yand body-size changes during growth showed: (a) equal or greater strength than the observed for body-size at birth, and (b) lower strength with respect to that obtained when considering current z-BMI at 6 y. In 6 y children variables capable of explaining CV variations showed a “hierarchical order”. Conversely, only z-BMI at 18 y showed significant associations with arterial z-scores at 18 y. Body size at birth showed almost no association with arterial characteristics at 6 or 18 y. The associations between ΔBWH z-score 0–2 y or Δz-BMI 0–6 y and CV properties at 6 y were mostly independent of body-size at birth. When current z-BMI was taken into account some associations between body changes in childhood and CV properties at 6 y were no longer significant. In adolescents, the associations between growth-related body changes and CV properties were dependent on z-BMI at the time of CVstudy.

Current z-BMI was the anthropometric parameter with the greatest capacity to explain the variations in CV properties at 6 y. However, interindividual variations in some hemodynamic and arterial parameters were mainly explained by growth-related anthropometric changes and/or by their interaction with current z-BMI. Similar findings were observed when the associations were analyzed taking into account the exposure to factors associated with CV risk. Current z-BMI at 18 y was the anthropometric variable with the greatest capacity to explain CV variations at 18 y. Body-size changes during childhood and/or adolescence contributed to explain arterial variations through the interaction with current z-BMI or BWH z-score at birth.

## Figures and Tables

**Table 1:** Clinical, anthropometric and arterial parameters, of children and adolescent cohorts.

	Children Cohort (*n* = 632)						Adolescent Cohort (*n* = 340)					
	MV	STD	Min.	p25th	p75th	Max.	MV	STD	Min.	p25th	p75th	Max.
Demographic, anthropometric and cardiovascular risk factors												
Age (years)	6.02	0.3	5.07	5.80	6.26	6.66	18.2	0.50	17.0	17.8	18.5	20.7
Subjects (n, %female)	632 (49.5)						340 (53.2)					
Body weight (kg)	22.31	4.67	14.00	19.20	24.35	46.50	64.4	14.2	40.2	55.0	70.0	120.0
Body height (m)	1.14	0.05	0.99	1.10	1.17	1.33	1.66	0.09	1.32	1.60	1.73	1.94
BMI (Kg/m^2^)	17.03	2.48	12.10	15.37	18.06	27.15	23.17	4.47	15.1	20.2	24.7	48.2
z-BMI (STD)	0.96	1.47	−2.60	0.06	1.62	7.37	0.36	1.16	-3.4	−0.4	1.0	4.8
z-BW for age (STD)	0.45	1.30	−2.39	−0.45	1.18	6.15	-	-	-	-	-	-
z-BH for age (STD)	−0.33	1.04	−3.43	−1.05	0.29	3.55	−0.41	0.88	−4.73	-0.90	0.13	2.39
Obesity (n, %)	108 (17.1)						26 (7.4)					
Dyslipidemia (n, %)	1 (0.2)						18 (5.3)					
Diabetes (n, %)	1 (0.2)						0 (0.0)					
Hypertension (n, %)	27 (4.3)						18 (5.3)					
CV Family background (n, %)	0 (0.0)						13 (3.9)					
Current Smoke (n, %)	0 (0.0)						35 (10.4)					
Anthropometric z-scores												
z-BW for age at birth (STD)	0.02	0.95	−5.24	−0.56	0.68	2.95	−0.18	1.24	-5.19	−0.89	0.57	2.87
z-BH for age at birth (STD)	−0.27	1.13	−4.91	−1.00	0.46	4.75	−0.35	1.52	-5.75	−1.15	0.46	4.82
z-BMI for age at birth (STD)	0.27	1.06	−3.59	−0.38	0.93	3.65	−0.01	1.72	−6.31	−1.00	0.96	5.56
z-BW for height 24 m. (STD)	0.59	1.26	−3.86	−0.20	1.29	4.76	-	-	-	-	-	-
z-BW for age 24 m. (STD)	0.38	1.14	−2.83	−0.44	1.08	4.54	-	-	-	-	-	-
z-BH for age 24 m. (STD)	−0.15	1.11	−4.12	−0.92	0.60	3.30	-	-	-	-	-	-
z-BMI for age 24 m. (STD)	0.66	1.29	−4.07	−0.15	1.40	5.42	-	-	-	-	-	-
1st Wave: anthropometry												
Age (years)	-	-	-	-	-	-	6.7	0.5	5.42	6.32	6.92	8.98
z-BW for age (STD)	-	-	-	-	-	-	0.39	1.01	−1.93	−0.27	1.06	3.56
z-BH for age (STD)	-	-	-	-	-	-	−0.13	0.93	−3.19	−0.72	0.53	2.10
z-BMI for age (STD)	-	-	-	-	-	-	0.65	0.99	−1.81	−0.08	1.27	4.63
2nd Wave: anthropometry												
Age (years)	-	-	-	-	-	-	8.1	0.4	6.82	7.77	8.38	10.27
z-BW for age (STD)	-	-	-	-	-	-	0.44	1.14	−2.40	–0.36	1.24	4.05
z-BH for age (STD)	-	-	-	-	-	-	−0.10	0.98	−4.53	–0.69	0.51	2.07
z-BMI for age (STD)	-	-	-	-	-	-	0.66	1.16	−3.12	−0.17	1.36	4.99
3rd Wave: anthropometry												
Age (years)	-	-	-	-	-	-	12.2	0.5	10.74	11.83	12.46	14.80
z-BW for age (STD)	-	-	-	-	-	-	-	-	-	-	-	-
z-BH for age (STD)	-	-	-	-	-	-	–0.03	1.12	–3.46	–0.87	0.71	2.98
z-BMI for age (STD)	-	-	-	-	-	-	0.47	1.14	–3.17	–0.37	1.23	3.12
Aortic and peripheral blood pressure, hemodynamic and wave reflection parameters												
Heart rate (beats/min)	91	11	66	84	99	134	69	12	43	61	76	132
pSBP (mmHg)	100	8	80	94	105	126	120	11	90	113	127	156
pDBP (mmHg)	59	7	50	54	62	86	64	7	48	60	68	100
pPP (mmHg)	41	7	24	36	46	77	56	11	21	48	63	90
pMBP (mmHg)	72	7	31	68	77	96	83	7	65	78	87	118
cSBP (mmHg)	83	6	64	78	87	100	102	9	83	96	107	135
cPP (mmHg)	22	4	7	19	25	43	37	8	18	31	43	61
CO (liter/min)	4.4	0.3	3.2	4.1	4.8	5.7	5.4	0.7	3.9	4.9	5.9	7.1
C.I. (liter/min/m^2^)	4.9	0.6	3.5	4.4	5.4	5.9	3.2	0.8	1.8	2.9	3.5	16.0
SVR (s.mmHg/mL)	1.10	0.10	0.86	1.04	1.19	1.41	1.05	0.15	0.78	0.93	1.16	1.47
AIx (%)	9.7	9.7	−16	3	17	37	–0.7	9.1	–28.0	–6	5	25
AIx@75 (%)	17.0	9.3	−10	11	23	43	–3.3	10.0	–34.0	–10	2	27
AP (mmHg)	2	2	−5	1	4	9	–1	4	–15	–2	2	8
PF (mmHg)	20	5	7	17	23	43	36	10	9	29	42	82
PB (mmHg)	10	4	3	9	11	78	14	3	4	12	16	26
Structural arterial parameters, local and regional arterial stiffness												
R-CCA SD (mm)	6.04	0.50	4.83	5.70	6.39	7.41	6.82	0.51	5.20	6.48	7.11	8.82
R-CCA DD (mm)	5.38	0.47	4.21	5.03	5.71	6.91	6.16	0.47	4.53	5.88	6.46	7.90
R-CCA IMT (mm)	0.421	0.028	0.370	0.405	0.431	0.537	0.494	0.039	0.380	0.473	0.512	0.631
L-CCA SD (mm)	5.92	0.46	4.84	5.59	6.22	7.48	6.75	0.46	5.46	6.41	7.07	8.18
L-CCA DD (mm)	5.25	0.43	4.25	4.96	5.53	6.99	6.11	0.44	4.90	5.76	6.40	7.56
L-CCA IMT (mm)	0.420	0.027	0.316	0.405	0.431	0.567	0.492	0.042	0.343	0.472	0.508	0.722
R-CFA SD (mm)	4.74	0.53	3.44	4.42	5.05	6.50	7.20	0.99	5.42	6.44	7.94	9.76
R-CFA DD (mm)	4.42	0.51	3.13	4.11	4.72	6.11	6.87	0.93	5.22	6.13	7.52	9.44
R-CFA IMT (mm)	0.331	0.032	0.282	0.313	0.346	0.411	0.409	0.063	0.320	0.373	0.448	0.645
L-CFA SD (mm)	4.72	0.50	3.51	4.36	5.01	6.37	7.30	1.02	4.69	6.48	8.08	10.26
L-CFA DD (mm)	4.40	0.50	3.29	4.06	4.71	5.98	6.97	0.98	4.48	6.18	7.68	9.75
L-CFA IMT (mm)	0.335	0.026	0.271	0.319	0.355	0.398	0.393	0.060	0.313	0.355	0.417	0.585
R-CCA EM (mmHg)	187	45	71	156	216	304	360	89	181	292	416	643
L-CCA EM (mmHg)	179	46	54	146	205	314	358	88	186	300	397	670
R-CFA EM (mmHg)	618	267	237	417	724	1555	1317	530	533	944	1565	3411
L-CFA EM (mmHg)	592	219	219	427	711	1561	1349	535	529	962	1639	3497
cfPWV (m/s)	4.81	0.76	2.88	4.28	5.25	7.72	6.10	0.75	4.02	5.61	6.57	8.20

MV: mean value. STD: standard deviation. Min., Max.: minimum and maximum values. z: z-score. p25th, p75th: percentile 25 and 75. BW, BH: body weight and height. BMI: body mass index. CV: cardiovascular. pSBP, pDBP, pPP, pMBP: peripheral systolic, diastolic, pulse and mean pressure. CO, C.I.: cardiac output and index. cSBP, cPP: central systolic and pulse pressure. SVR: systemic vascular resistances. AIx, AIx@75: aortic augmentation index without and with heart rate adjustment. AP: augmented pressure. PF and PB: forward and backward aortic pressure component. R: Right. L: Left. CCA, CFA: common carotid and femoral artery. DD, SD: diastolic and systolic diameter. EM: elastic modulus. IMT: intima-media thickness. cfPWV: carotid-femoral pulse wave velocity.

**Table 2:** Comparative analysis CV variables’ association with anthropometric characteristics at birth, at 6 y and with anthropometric changes within that period (0–2 and 0–6 y) (children cohort, *n* = 632).

	Zero-OrderCorrelations								William Test (Comparison of Correlations)				
	Δ z-BWH (0–2y) [1]		Δ z-BMI (0–6y) [2]		z-BWH (at Birth) [3]		z-BMI (at 6 y) [4]		1–2	1–3	1–4	2–3	2–4
	*R*	*p*	*R*	*p*	*R*	*p*	*R*	*p*	*p*	*p*	*P*	*p*	*p*
Aortic and peripheral blood pressure, hemodynamic and wave reflection parameters													
z-pSBP	0.16	0.026	0.20	0.003	0.08	0.228	0.32	<0.001	0.408	0.469	0.019	0.245	0.009
z-pDBP	0.13	0.064	0.16	0.019	0.05	0.437	0.22	<0.001	0.538	0.476	0.182	0.299	0.174
z-pPP	0.05	0.474	0.06	0.389	0.03	0.613	0.13	0.034	0.839	0.883	0.236	0.805	0.110
z-pMBP	0.16	0.027	0.19	0.005	0.07	0.304	0.28	<0.001	0.514	0.425	0.062	0.243	0.038
z-cSBP	0.18	0.008	0.20	0.003	0.05	0.391	0.29	<0.001	0.663	0.253	0.079	0.138	0.042
z-cDBP	0.14	0.040	0.16	0.017	0.04	0.501	0.23	<0.001	0.665	0.355	0.150	0.237	0.091
z-cPP	0.06	0.396	0.07	0.312	0.04	0.579	0.10	0.106	0.831	0.831	0.549	0.726	0.534
z-AIx	–0.16	0.017	–0.17	0.009	0.11	0.078	–0.16	0.009	0.828	0.008	0.964	0.005	0.762
z-AIx@75	–0.16	0.020	–0.16	0.014	0.03	0.648	–0.23	<0.001	0.949	0.073	0.291	0.063	0.106
z-AP	–0.15	0.027	–0.14	0.030	0.12	0.054	–0.11	0.064	0.829	0.009	0.576	0.008	0.502
z-Pf	0.11	0.092	0.13	0.053	–0.02	0.791	0.17	0.006	0.667	0.208	0.427	0.143	0.395
z-Pb	0.01	0.938	0.00	0.949	0.04	0.494	0.03	0.609	0.850	0.709	0.701	0.634	0.427
Structural arterial parameters, local and regional arterial stiffness													
z-R-CCA SD	0.08	0.455	0.24	0.016	0.03	0.785	0.29	0.001	0.026	0.758	0.036	0.168	0.430
z-R-CCA DD	0.07	0.506	0.22	0.024	0.03	0.744	0.29	0.002	0.002	0.820	0.033	0.225	0.311
z-R-CCA IMT	–0.29	0.004	0.28	0.005	–0.05	0.631	0.29	<0.001	<0.001	0.018	<0.001	0.001	0.833
z-L-CCA SD	0.12	0.092	0.15	0.027	0.13	0.039	0.19	0.037	0.532	0.911	0.476	0.923	0.545
z-L-CCA DD	0.09	0.206	0.14	0.035	0.16	0.012	0.29	<0.001	0.298	0.469	0.003	0.812	<0.001
z-L-CCA IMT	0.11	0.115	0.14	0.043	0.07	0.178	0.18	0.004	0.695	0.735	0.312	0.513	0.390
z-R-CFA SD	0.32	0.003	0.36	<0.001	–0.16	0.118	0.36	<0.001	0.807	0.004	0.708	0.001	0.952
z-R-CFA DD	0.34	0.001	0.35	<0.001	–0.18	0.077	0.33	<0.001	0.827	0.002	0.890	0.001	0.765
z-R-CFA IMT	–0.23	0.338	–0.25	0.296	0.14	0.552	–0.40	0.065	0.674	0.335	0.470	0.294	0.323
z-L-CFA SD	0.11	0.123	0.14	0.034	0.09	0.194	0.29	<0.001	0.767	0.838	0.086	0.610	<0.001
z-L-CFA DD	0.09	0.198	0.12	0.071	0.08	0.208	0.26	<0.001	0.690	0.948	0.014	0.730	0.002
z-L-CFA IMT	0.08	0.617	0.17	0.250	–0.12	0.379	–0.01	0.919	0.101	0.372	0.542	0.173	0.001
z-R-CCA EM	0.05	0.620	0.12	0.226	–0.02	0.822	0.13	0.162	0.205	0.650	0.463	0.028	0.864
z-L-CCA EM	–0.03	0.746	0.06	0.405	0.11	0.161	0.12	0.080	0.242	0.280	0.057	0.697	0.213
z-R-CFA EM	0.15	0.179	0.07	0.493	–0.08	0.422	0.00	0.981	0.103	0.177	0.189	0.353	0.343
z-L-CFA EM	–0.04	0.596	–0.06	0.407	0.04	0.579	–0.01	0.863	0.115	0.993	0.703	0.360	0.287
z-cfPWV	0.00	0.991	–0.03	0.775	–0.02	0.790	0.00	1.00	0.649	0.876	0.989	0.959	0.610

z: z-score. BW, BH: body weight and height. Δ: change in the analyzed period. BMI: body mass index. SBP, DBP, PP, MBP: systolic, diastolic, pulse and mean pressure (p: peripheral, c: central). AIx, AIx@75: aortic augmentation index without and with heart rate adjustment. AP: augmented pressure. Pf, Pb: forward and backward pressure components. R: Right. L: Left. CCA, CFA: common carotid and femoral artery. EM: elastic modulus. IMT: intima-media thickness. DD, SD: diastolic and systolic diameter. cfPWV: carotid-femoral pulse wave velocity. *R*: Pearson coefficient. *p <* 0.05 (red text) was considered significant. In Zero-order correlations columns, the numbers in square brackets define de groups. Then in "William Test (Comparison of Correlations)" (also in columns in the same table), groups are compared in pairs cnsidering groups numbers. Identical comment for next Tables.

**Table 3:** Comparative analysis CV variables’ association with anthropometric characteristics at birth, at 18 y and with anthropometric changes within that period (0–6, 0–18, 6–18 y) [adolescent cohort, *n* = 340].

	Zero-Ordercorrelations										William Test (Comparison of Correlations)								
	Δ z-BMI (0–6y) [1]		Δ z-BMI (0–18y) [2]		Δ z-BMI (6–18y) [3]		z-BWH (at Birth) [4]		z-BMI (18y) [5]		1–2	1–3	1–4	1–5	2–3	2–4	2–5	3–4	3–5
	*R*	*p*	*R*	*p*	*R*	*p*	*R*	*p*	*R*	*p*	*p*	*p*	*p*	*p*	*p*	*p*	*p*	*p*	*p*
Aortic and peripheral blood pressure, hemodynamic and reflection parameters																			
z-pSBP	0.08	0.234	0.14	0.021	0.09	0.172	0.01	0.931	0.25	<0.001	0.411	0.905	0.488	0.010	0.421	0.203	0.040	0.458	0.009
z-pDBP	−0.02	0.757	0.02	0.798	0.07	0.286	0.03	0.581	0.17	0.002	0.623	0.283	0.609	0.004	0.424	0.922	0.006	0.711	0.107
z-pPP	0.10	0.111	0.14	0.016	0.04	0.478	−0.02	0.715	0.15	0.007	0.541	0.473	0.002	0.460	0.108	0.118	0.854	0.579	0.077
z-pMBP	0.02	0.732	0.08	0.206	0.09	0.162	0.03	0.660	0.24	<0.001	0.438	0.403	0.954	0.001	0.873	0.283	0.003	0.577	0.014
z-cSBP	0.02	0.765	0.07	0.226	0.05	0.461	0.10	0.081	0.27	<0.001	0.464	0.721	0.421	<0.001	0.749	0.775	<0.001	0.641	0.000
z-cDBP	0.002	0.977	0.03	0.613	−0.01	0.818	0.09	0.135	0.14	0.011	0.692	0.886	0.396	0.038	0.523	0.568	0.050	0.353	0.016
z-cPP	0.02	0.773	0.05	0.394	0.06	0.301	0.04	0.504	0.18	0.001	0.663	0.634	0.844	0.017	0.873	0.924	0.020	0.853	0.053
z-AIx	0.02	0.744	0.02	0.682	0.03	0.602	−0.01	0.931	−0.02	0.774	0.944	0.906	0.495	0.008	0.874	0.772	0.471	0.714	0.428
z-AIx@75	−0.05	0.416	0.004	0.948	0.09	0.159	0.07	0.265	0.01	0.918	0.451	0.287	0.266	0.110	0.172	0.523	0.970	0.853	0.204
z-AP	0.02	0.735	0.02	0.741	0.02	0.744	0.001	0.996	−0.02	0.693	1.000	0.987	0.850	<0.001	0.992	0.849	0.470	0.398	0.526
z-PF	0.06	0.397	0.06	0.308	0.02	0.707	−0.02	0.755	0.11	0.047	0.967	0.643	0.459	0.432	0.535	0.449	0.376	0.719	0.161
z-PB	0.11	0.096	0.06	0.309	−0.08	0.211	0.05	0.402	0.06	0.289	0.519	0.028	0.578	0.484	0.030	0.925	0.990	0.243	0.030
Structural arterial parameters, local and regional arterial stiffness																			
z-R-CCA SD	0.09	0.146	0.15	0.015	0.10	0.123	−0.02	0.692	0.26	<0.001	0.437	0.905	0.273	0.012	0.423	0.098	0.041	0.269	0.009
z-R-CCA DD	0.08	0.208	0.14	0.021	0.09	0.129	−0.003	0.962	0.26	<0.001	0.413	0.905	0.291	0.006	0.424	0.164	0.026	0.392	0.006
z-R-CCA IMT	0.04	0.501	0.03	0.604	0.12	0.060	0.002	0.967	0.12	0.032	0.900	0.341	0.716	0.238	0.153	0.787	0.104	0.277	0.949
z-L-CCA SD	0.11	0.077	0.13	0.034	0.10	0.100	−0.01	0.883	0.25	<0.001	0.811	0.905	0.248	0.034	0.632	0.174	0.026	0.312	0.015
z-L-CCA DD	0.10	0.121	0.12	0.054	0.09	0.136	0.03	0.597	0.28	<0.001	0.822	0.905	0.513	0.007	0.632	0.382	0.003	0.581	0.002
z-L-CCA IMT	0.12	0.069	0.06	0.283	0.09	0.150	−0.01	0.868	0.13	0.020	0.440	0.721	0.212	0.860	0.634	0.772	0.205	0.359	0.524
z-R-CFA SD	0.12	0.233	0.08	0.418	0.18	0.057	0.02	0.840	0.34	<0.001	0.708	0.647	0.537	0.036	0.309	0.712	0.002	0.345	0.133
z-R-CFA DD	0.11	0.281	0.08	0.437	0.18	0.069	0.01	0.957	0.32	<0.001	0.755	0.593	0.522	0.046	0.309	0.667	0.004	0.316	0.140
z-R-CFA IMT	0.15	0.496	0.17	0.390	0.19	0.363	−0.16	0.423	0.27	0.149	0.936	0.886	0.380	0.607	0.924	0.343	0.582	0.339	0.697
z-L-CFA SD	0.11	0.292	0.11	0.244	0.11	0.273	−0.02	0.870	0.26	0.003	0.986	0.988	0.440	0.148	0.960	0.422	0.077	0.446	0.120
z-L-CFA DD	0.09	0.407	0.10	0.307	0.10	0.322	0.01	0.951	0.26	0.003	0.943	0.940	0.607	0.100	0.984	0.578	0.059	0.597	0.097
z-L-CFA IMT	0.01	0.967	−0.15	0.445	−0.26	0.190	0.27	0.162	0.33	0.068	0.160	0.328	0.437	0.151	0.589	0.215	0.005	0.136	0.002
z-R-CCA EM	−0.05	0.485	0.02	0.788	0.06	0.317	0.12	0.043	0.21	<0.001	0.360	0.196	0.102	<0.001	0.528	0.327	0.003	0.581	0.016
z-L-CCA EM	0.02	0.787	0.06	0.296	0.06	0.345	0.13	0.029	0.31	<0.001	0.551	0.639	0.288	<0.001	0.954	0.498	0.003	0.520	<0.001
z-R-CFA EM	−0.03	0.793	0.02	0.821	−0.10	0.308	−0.09	0.364	−0.07	0.443	0.648	0.601	0.725	0.721	0.230	0.503	0.306	0.953	0.764
z-L-CFA EM	−0.14	0.175	−0.05	0.630	−0.09	0.351	0.12	0.223	0.09	0.309	0.408	0.706	0.118	0.032	0.689	0.298	0.109	0.222	0.070
z-cfPWV	0.01	0.912	0.01	0.802	−0.03	0.621	0.03	0.632	0.03	0.599	0.944	0.633	0.862	0.770	0.523	0.845	0.716	0.579	0.337

z: z-score. BW, BH: body weight and height. Δ: change in the studied period. BMI: body mass index. SBP, DBP, PP, MBP: systolic, diastolic, pulse and mean pressure (p: peripheral, c: central). AIx, AIx@75: augmentation index without and with heart rate adjustment. AP: augmented pressure. Pf, Pb: forward and backwardpressure components. R: Right. L: Left. CCA, CFA: common carotid and femoral artery. EM: elastic modulus. IMT: intima-media thickness. DD, SD: diastolic and systolic diameter. cfPWV: carotid-femoral pulse wave velocity. R: Pearson coefficient. *p <* 0.05 (red) was considered significant.

**Table 4:** Comparative analysis of the associations between CV and anthropometric parameters: children vs. adolescents.

	Zero-OrderCorrelations										Comparison of Correlations					
	ChildrenCohort (*n* = 632)				AdolescentCohort (*n* = 340)											
	ΔBWH (0–2y) [1]		Δ z-BMI (0–6y) [2]		Δ z-BMI (0–6y) [3]		Δ z-BMI (0–18y) [4]		Δ z-BMI (6–18y) [5]		1–3	1–4	1–5	2–3	2–4	2–5
	*R*	*p*	*R*	*p*	*R*	*p*	*R*	*p*	*R*	*p*	*p*	*P*	*p*	*P*	*p*	*p*
Aortic and peripheral blood pressure, hemodynamic and wave reflection parameters																
z-pSBP	0.16	0.026	0.20	0.003	0.08	0.234	0.14	0.021	0.09	0.172	0.320	0.802	0.384	0.613	0.449	0.169
z-pDBP	0.13	0.064	0.16	0.019	−0.02	0.757	0.02	0.798	0.07	0.286	0.065	0.175	0.458	0.708	0.084	0.264
z-pPP	0.05	0.474	0.06	0.389	0.10	0.111	0.14	0.016	0.04	0.478	0.538	0.266	0.624	0.902	0.322	0.806
z-pMBP	0.16	0.027	0.19	0.005	0.02	0.732	0.08	0.206	0.09	0.162	0.083	0.320	0.383	0.705	0.170	0.211
z-cSBP	0.18	0.008	0.20	0.003	0.02	0.765	0.07	0.226	0.05	0.461	0.047	0.159	0.097	0.794	0.095	0.055
z-cDBP	0.14	0.040	0.16	0.017	0.002	0.977	0.03	0.613	−0.01	0.818	0.080	0.163	0.058	0.797	0.098	0.031
z-cPP	0.06	0.396	0.07	0.312	0.02	0.773	0.05	0.394	0.06	0.301	0.614	0.900	0.930	0.899	0.801	0.899
z-AIx	−0.16	0.017	−0.17	0.009	0.02	0.744	0.02	0.682	0.03	0.602	0.023	0.023	0.016	0.897	0.016	0.011
z-AIx@75	−0.16	0.020	−0.16	0.014	−0.05	0.416	0.004	0.948	0.09	0.159	0.169	0.041	0.002	0.962	0.041	0.002
z-AP	−0.15	0.027	−0.14	0.030	0.02	0.735	0.02	0.741	0.02	0.744	0.035	0.032	0.032	0.898	0.043	0.043
z-Pf	0.11	0.092	0.13	0.053	0.06	0.397	0.06	0.308	0.02	0.707	0.534	0.534	0.263	0.802	0.383	0.063
z-Pb	0.01	0.938	−0.004	0.949	0.11	0.096	0.06	0.309	−0.08	0.211	0.215	0.537	0.266	0.883	0.429	0.347
Structural arterial parameters, local and regional arterial stiffness																
z-R-CCA SD	0.08	0.455	0.24	0.016	0.09	0.146	0.15	0.015	0.10	0.123	0.924	0.503	0.248	0.121	0.377	0.173
z-R-CCA DD	0.07	0.506	0.22	0.024	0.08	0.208	0.14	0.021	0.09	0.129	0.924	0.404	0.849	0.148	0.435	0.208
z-R-CCA IMT	−0.29	0.004	0.28	0.005	0.04	0.501	0.03	0.604	0.12	0.060	0.001	0.002	<0.001	<0.001	0.015	0.115
z-L-CCA SD	0.12	0.092	0.15	0.027	0.11	0.077	0.13	0.034	0.10	0.100	0.900	0.900	0.803	0.706	0.801	0.530
z-L-CCA DD	0.09	0.206	0.14	0.035	0.10	0.121	0.12	0.054	0.09	0.136	0.901	0.708	0.940	0.531	0.802	0.531
z-L-CCA IMT	0.11	0.115	0.14	0.043	0.12	0.069	0.06	0.283	0.09	0.150	0.900	0.535	0.803	0.708	0.318	0.531
z-R-CFA SD	0.32	0.003	0.36	<0.001	0.12	0.233	0.08	0.418	0.18	0.057	0.100	0.050	0.244	0.725	0.021	0.129
z-R-CFA DD	0.34	0.001	0.35	0.001	0.11	0.281	0.08	0.437	0.18	0.069	0.058	0.033	0.180	0.930	0.026	0.153
z-R-CFA IMT	−0.23	0.338	−0.25	0.296	0.15	0.496	0.17	0.390	0.19	0.363	0.173	0.151	0.131	0.942	0.131	0.113
z-L-CFA SD	0.11	0.123	0.14	0.034	0.11	0.292	0.11	0.244	0.11	0.273	0.992	0.977	0.992	0.773	0.773	0.773
z-L-CFA DD	0.09	0.198	0.12	0.071	0.09	0.407	0.10	0.307	0.10	0.322	0.970	0.924	0.924	0.774	0.848	0.848
z-L-CFA IMT	0.08	0.617	0.17	0.250	0.01	0.967	−0.15	0.445	−0.26	0.190	0.751	0.296	0.118	0.679	0.145	0.048
z-R-CCA EM	0.05	0.620	0.12	0.226	−0.05	0.485	0.02	0.788	0.06	0.317	0.355	0.781	0.926	0.515	0.353	0.576
z-L-CCA EM	−0.03	0.746	0.06	0.405	0.02	0.787	0.06	0.296	0.06	0.345	0.570	0.306	0.306	0.306	0.991	0.955
z-R-CFA EM	0.15	0.179	0.07	0.493	−0.03	0.793	0.02	0.821	−0.10	0.308	0.160	0.309	0.051	0.530	0.698	0.186
z-L-CFA EM	−0.04	0.596	−0.06	0.407	−0.14	0.175	−0.05	0.630	−0.09	0.351	0.343	0.925	0.637	0.851	0.925	0.777
z-cfPWV	−0.001	0.991	−0.03	0.775	0.01	0.912	0.01	0.802	−0.03	0.621	0.908	0.9082	0.761	0.761	0.753	0.95

z: z-score. BW, BH: body weight and height. Δ: change in the analyzed period. BMI: body mass index. SBP, DBP, PP, MBP: systolic, diastolic, pulse and mean pressure (p: peripheral, c: central). AIx, AIx@75: aortic augmentation index without and with heart rate adjustment. AP: augmented pressure. Pf, Pb: forward and backward pressure components. R: Right. L: Left. CCA, CFA: common carotid and femoral artery. EM: elastic modulus. IMT: intima-media thickness. DD, SD: diastolic and systolic diameter. cfPWV: carotid-femoral pulse wave velocity. *R*: Pearson coefficient. *p <* 0.05 (red) was considered significant.

**Table 5:** Associations between CV z-scores and z-BWHvariations between 0–2y (childrencohort, *n* = 632).

	Δ z-BWH (0–2y)					
	Zero-Order		Partial ^1^		Partial ^2^	
	*R*	*p*	*R*	*p*	*R*	*p*
Aortic and peripheral blood pressure, hemodynamic and wave reflection parameters						
z-pSBP	0.16	0.026	0.26	<0.001	0.11	0.103
z-pDBP	0.13	0.064	0.21	0.003	0.10	0.136
z-pPP	0.05	0.474	0.09	0.197	0.02	0.756
z-pMBP	0.16	0.027	0.25	<0.001	0.12	0.082
z-cSBP	0.18	0.008	0.27	<0.001	0.14	0.041
z-cDBP	0.14	0.040	0.21	0.002	0.10	0.136
z-cPP	0.06	0.396	0.10	0.135	0.06	0.379
z-AIx	−0.16	0.017	−0.12	0.085	−0.02	0.797
z-AIx@75	−0.16	0.020	−0.18	0.009	−0.06	0.419
z-AP	−0.15	0.027	−0.09	0.167	−0.02	0.762
z-Pf	0.11	0.092	0.13	0.052	0.04	0.528
z-Pb	0.01	0.938	0.04	0.545	0.03	0.619
Structural arterial parameters, local and regional arterial stiffness						
z-R-CCA SD	0.08	0.455	0.12	0.250	−0.06	0.595
z-R- CCA DD	0.07	0.506	0.11	0.277	−0.06	0.569
z-R-CCA IMT	−0.29	0.004	0.34	0.001	0.27	0.008
z-L-CCA SD	0.12	0.092	0.26	<0.001	0.13	0.058
z-L-CCA DD	0.09	0.206	0.24	<0.001	0.12	0.088
z-L-CCA IMT	0.11	0.115	0.20	0.004	0.13	0.048
z-R-CFA SD	0.32	0.003	0.28	0.008	0.08	0.472
z-R-CFA DD	0.34	0.001	0.30	0.005	0.12	0.288
z-R-CFA IMT	−0.23	0.338	−0.19	0.451	0.07	0.777
z-L-CFA SD	0.11	0.123	0.21	0.003	0.07	0.353
z-L-CFA DD	0.09	0.198	0.18	0.009	0.05	0.453
z-L-CFA IMT	0.08	0.617	0.00	0.991	−0.01	0.956
z-R-CCA EM	0.05	0.620	0.05	0.644	−0.03	0.750
z-L-CCA EM	−0.03	0.746	0.05	0.528	−0.01	0.888
z-R-CFA EM	0.15	0.179	0.12	0.270	0.13	0.223
z-L-CFA EM	−0.04	0.596	−0.02	0.796	−0.01	0.888
z-cfPWV	0.00	0.991	−0.02	0.827	−0.03	0.766

z: z-score. BW, BH: body weight and height. BMI: body mass index. SBP, DBP, PP, MBP: systolic, diastolic, pulse and mean pressure (p: peripheral, c: central). AIx, AIx@75: aortic augmentation index without and with heart rate adjustment. AP: augmented pressure. Pf, Pb: forward and backward pressure components. CCA, CFA: common carotid and femoral artery. EM: elastic modulus. IMT: intima-media thickness. DD, SD: diastolic and systolic diameter. cfPWV: carotid-femoral pulse wave velocity. *R*: Pearson coefficient. *p <* 0.05 (red) was considered significant. Partial correlations controlling for: ^1^ BW-for-BH (length) z-score at birth; ^2^ BW-for-BH (length) z-score at birth and z-BMI at the time of measurement (6 y).

**Table 6:** Associations between CV z-scores and z-BMIvariations between 0–6 y(both cohorts).

	Δ z-BMI (0–6y)													
	ChildrenCohort (*n* = 632)						AdolescentCohort (*n* = 340)							
	Zero-order		Partial ^1^		Partial ^2^		Zero-order		Partial ^1^		Partial ^2^		Partial ^3^	
	*R*	*p*	*R*	*p*	*R*	*p*	*R*	*p*	*R*	*p*	*R*	*p*	*R*	*p*
Aortic and peripheral blood pressure, hemodynamic and wave reflection parameters														
z-pSBP	0.20	**0.003**	0.28	**<0.001**	−0.04	0.588	0.08	0.234	0.13	**0.049**	0.02	0.805	0.18	**0.008**
z-pDBP	0.16	**0.019**	0.22	**0.001**	0.03	0.621	−0.02	0.757	0.01	0.890	−0.08	0.239	0.04	0.558
z-pPP	0.06	0.389	0.09	0.195	−0.11	**0.095**	0.10	0.111	0.14	**0.037**	0.08	0.245	0.17	**0.013**
z-pMBP	0.19	**0.005**	0.26	**<0.001**	0.00	0.961	0.02	0.732	0.07	0.302	−0.05	0.483	0.11	**0.097**
z-cSBP	0.20	**0.003**	0.26	**<0.001**	−0.04	0.529	0.02	0.765	0.16	0.014	0.05	0.483	0.20	**0.003**
z-cDBP	0.16	**0.017**	0.21	**0.001**	−0.04	0.567	0.00	0.977	0.11	**0.083**	0.06	0.380	0.12	**0.074**
z-cPP	0.07	0.312	0.10	0.135	0.03	0.624	0.02	0.773	0.08	0.228	0.00	0.977	0.11	**0.088**
z-cMBP	0.18	**0.005**	0.24	**<0.001**	−0.02	0.718	−0.04	0.556	−0.03	0.636	−0.09	0.158	0.06	0.406
z-AIx	−0.17	**0.009**	−0.13	**0.045**	0.14	**0.034**	0.02	0.744	0.03	0.676	0.04	0.555	0.04	0.519
z-AIx@75	−0.16	**0.014**	−0.17	**0.009**	0.19	**0.005**	−0.05	0.416	0.00	0.984	0.00	0.976	0.04	0.562
z-AP	−0.14	**0.030**	−0.09	0.161	0.12	**0.056**	0.02	0.735	0.04	0.590	0.05	0.439	0.05	0.485
z-Pf	0.13	**0.053**	0.14	0.036	−0.09	0.173	0.06	0.397	0.07	0.322	0.02	0.820	0.08	0.228
z-Pb	0.00	0.949	0.02	0.747	0.00	0.956	0.11	**0.096**	0.24	**<0.001**	0.24	**<0.001**	0.23	**0.001**
Structural arterial parameters, local and regional arterial stiffness														
z-R-CCA SD	0.24	**0.016**	0.29	**0.003**	0.05	0.639	0.09	0.146	0.12	**0.066**	0.00	0.968	0.17	**0.011**
z-R-CCA DD	0.22	**0.024**	0.28	**0.005**	0.02	0.838	0.08	0.208	0.13	**0.054**	0.01	0.917	0.18	**0.008**
z-R-CCA IMT	0.28	**0.005**	0.29	**0.003**	0.33	**0.001**	0.04	0.501	0.07	0.268	0.02	0.763	0.13	**0.054**
z-L-CCA SD	0.15	**0.027**	0.25	**<0.001**	−0.02	0.779	0.11	**0.077**	0.17	**0.009**	0.06	0.321	0.23	**0.001**
z-L-CCA DD	0.14	**0.035**	0.26	**<0.001**	0.03	0.623	0.10	0.121	0.20	**0.002**	0.09	0.189	0.26	**<0.001**
z-L-CCA IMT	0.14	**0.043**	0.20	**0.002**	0.13	**0.044**	0.12	**0.069**	0.17	**0.007**	0.13	**0.048**	0.24	**<0.001**
z-R-CFA SD	0.36	**<0.001**	0.33	**0.002**	−0.17	0.105	0.12	0.233	0.22	**0.032**	0.08	0.428	0.32	**0.002**
z-R-CFA DD	0.35	**0.001**	0.31	**0.003**	−0.18	**0.094**	0.11	0.281	0.18	**0.075**	0.05	0.645	0.28	**0.007**
z-R-CFA IMT	−0.25	0.296	−0.21	0.397	0.74	**<0.001**	0.15	0.496	0.04	0.865	−0.10	0.645	0.12	0.607
z-L-CFA SD	0.14	**0.034**	0.22	**0.001**	−0.15	**0.033**	0.11	0.292	0.16	0.136	0.04	0.685	0.22	**0.039**
z-L-CFA DD	0.12	**0.071**	0.19	**0.004**	−0.15	**0.032**	0.09	0.407	0.15	0.164	0.03	0.769	0.20	**0.056**
z-L-CFA IMT	0.17	0.250	0.12	0.413	0.36	**0.013**	0.01	0.967	0.36	**0.073**	0.26	0.218	0.30	0.149
z-R-CCA EM	0.12	0.226	0.13	0.203	0.00	0.977	−0.05	0.485	0.08	0.229	−0.01	0.850	0.12	0.080
z-L-CCA EM	0.06	0.405	0.14	**0.073**	0.12	0.130	0.02	0.787	0.19	**0.003**	0.07	0.320	0.24	**<0.001**
z-R-CFA EM	0.07	0.493	0.04	0.741	0.06	0.581	−0.03	0.793	−0.15	0.144	−0.14	0.184	−0.21	**0.042**
z-L-CFA EM	−0.06	0.407	−0.04	0.524	−0.09	0.214	−0.14	0.175	−0.08	0.436	−0.14	0.205	−0.12	0.241
z-cfPWV	−0.03	0.775	−0.04	0.625	−0.15	**0.083**	0.01	0.912	0.05	0.475	0.04	0.560	0.04	0.566

z: z-score. BMI: body mass index. SBP, DBP, PP, MBP: systolic, diastolic, pulse and mean pressure (p: peripheral, c: central). AIx, AIx@75: augmentation index without and with heart rate adjustment. AP: augmented pressure. Pf,Pb: forward and backward pressure components. R: Right. L: Left. CCA, CFA: common carotid and femoral artery. EM: elastic modulus. IMT: intima-media thickness. DD, SD: diastolic and systolic diameter. cfPWV: carotid-femoral pulse wave velocity. *R*: Pearson coefficient. *p <* 0.05 (red text) was considered significant. Partial correlations controlling for: ^1^ BW-for-BHz-score at birth; ^2^ BWH z-score at birth and z-BMI at the time of CV study; ^3^ BWH z-score at birth and z-BMI variation between 6 and 18 y.

**Table 7:** Associations between CVz-scores and z-BMI variations between 0–18y(adolescent cohort, *n* = 340).

	Δ z-BMI (0–18y)					
	Zero-Order		Partial ^1^		Partial ^2^	
	*R*	*P*	*R*	*p*	*R*	*p*
Aortic and peripheral blood pressure, hemodynamic and wave reflection parameters						
z-pSBP	0.14	0.021	−0.14	0.682	−0.19	0.595
z-pDBP	0.02	0.798	−0.03	0.923	0.37	0.291
z-pPP	0.14	0.016	−0.13	0.712	−0.73	0.017
z-pMBP	0.08	0.206	−0.07	0.836	0.21	0.558
z-cSBP	0.07	0.226	0.27	0.419	0.31	0.388
z-cDBP	0.03	0.613	−0.10	0.777	0.02	0.962
z-cPP	0.05	0.394	0.61	0.045	0.57	0.084
z-AIx	0.02	0.682	−0.06	0.851	0.23	0.527
z-AIx@75	0.00	0.948	−0.13	0.704	0.13	0.720
z-AP	0.02	0.741	−0.08	0.818	0.16	0.668
z-Pf	0.06	0.308	0.05	0.895	0.16	0.660
z-Pb	0.06	0.309	0.15	0.658	0.15	0.679
Structural arterial parameters, local and regional arterial stiffness						
z-R-CCA SD	0.15	0.015	0.07	0.846	0.23	0.517
z-R-CCA DD	0.14	0.021	0.04	0.902	0.25	0.494
z-R-CCA IMT	0.03	0.604	0.15	0.667	−0.26	0.468
z-L-CCA SD	0.13	0.034	0.11	0.755	0.07	0.844
z-L-CCA DD	0.12	0.054	0.08	0.820	0.10	0.789
z-L-CCA IMT	0.06	0.283	−0.05	0.895	0.06	0.878
z-R-CFA SD	0.08	0.418	0.01	0.967	0.20	0.575
z-R-CFA DD	0.08	0.437	−0.08	0.806	0.18	0.615
z-R-CFA IMT	0.17	0.390	0.31	0.354	−0.16	0.656
z-L-CFA SD	0.11	0.244	−0.02	0.948	0.12	0.750
z-L-CFA DD	0.10	0.307	−0.17	0.622	0.10	0.784
z-L-CFA IMT	−0.15	0.445	0.08	0.813	0.13	0.731
z-R-CCA EM	0.02	0.788	0.38	0.254	0.33	0.359
z-L-CCA EM	0.06	0.296	0.37	0.267	0.44	0.208
z-R-CFA EM	0.02	0.821	−0.31	0.358	−0.37	0.298
z-L-CFA EM	−0.05	0.630	−0.52	0.105	−0.29	0.409
z-cfPWV	0.01	0.802	−0.29	0.387	0.32	0.367

z: z-score. BW, BH: body weight and height. BMI: body mass index. SBP, DBP, PP, MBP: systolic, diastolic, pulse and mean pressure (p: peripheral, c: central). AIx, AIx@75: augmentation index without and with heart rate adjustment. AP: augmented pressure. Pf, Pb: forward and backward pressure components. CCA, CFA: common carotid and femoral artery. EM: elastic modulus. IMT: intima-media thickness. DD, SD: diastolic and systolic diameter. cfPWV: carotid-femoral pulse wave velocity. *R*: Pearson coefficient. *p <* 0.05 (red) was considered significant. Partial correlations controlling for: ^1^ BW-for-BH (length) z-score at birth; ^2^ BW-for-BH z-score at birth and z-BMI at the time of CV study.

**Table 8:** Associations between CV z-scores and z-BMI variations between 6–18 y (adolescent cohort, *n* = 340).

	Δ z-BMI (6–18y)					
	Zero-Order		Partial ^1^		Partial ^2^	
	*R*	*p*	*R*	*p*	*R*	*p*
Aortic and peripheral blood pressure, hemodynamic and wave reflection parameters						
z-pSBP	0.09	0.172	−0.29	0.453	−0.24	0.566
z-pDBP	0.07	0.286	−0.31	0.419	−0.23	0.591
z-pPP	0.04	0.478	0.04	0.918	0.01	0.980
z-pMBP	0.09	0.162	−0.35	0.357	−0.27	0.521
z-cSBP	0.05	0.461	−0.22	0.565	−0.65	**0.079**
z-cDBP	−0.01	0.818	−0.42	0.260	−0.42	0.305
z-cPP	0.06	0.301	0.12	0.749	−0.51	0.193
z-AIx	0.03	0.602	−0.56	0.114	−0.51	0.200
z-AIx@75	0.09	0.159	−0.53	0.142	−0.45	0.264
z-AP	0.02	0.744	−0.56	0.114	−0.49	0.219
z-PF	0.02	0.707	−0.14	0.725	−0.31	0.453
z-PB	−0.08	0.211	−0.20	0.598	−0.37	0.364
Structural arterial parameters, local and regional arterial stiffness						
z-R-CCA SD	0.10	0.123	−0.55	0.123	−0.74	**0.035**
z-R-CCA DD	0.09	0.129	−0.38	0.308	−0.59	0.120
z-R-CCA IMT	0.12	**0.060**	0.20	0.600	−0.14	0.747
z-L-CCA SD	0.10	0.100	−0.18	0.651	−0.23	0.583
z-L-CCA DD	0.09	0.136	−0.10	0.804	−0.11	0.791
z-L-CCA IMT	0.09	0.150	−0.01	0.975	−0.33	0.418
z-R-CFA SD	0.18	**0.057**	0.14	0.722	0.13	0.754
z-R-CFA DD	0.18	**0.069**	0.10	0.795	0.30	0.475
z-R-CFA IMT	0.19	0.363	0.28	0.473	−0.34	0.415
z-L-CFA SD	0.11	0.273	−0.04	0.927	−0.14	0.748
z-L-CFA DD	0.10	0.322	−0.11	0.776	0.01	0.988
z-L-CFA IMT	−0.26	0.190	−0.28	0.470	−0.52	0.184
z-R-CCA EM	0.06	0.317	0.26	0.503	−0.20	0.631
z-L-CCA EM	0.06	0.345	0.13	0.746	−0.21	0.619
z-R-CFA EM	−0.10	0.308	0.12	0.759	0.66	**0.075**
z-L-CFA EM	−0.09	0.351	−0.20	0.604	0.37	0.365
z-cfPWV	−0.03	0.621	−0.63	**0.068**	−0.35	0.390

z: z-score. BW, BH: body weight and height. BMI: body mass index. SBP, DBP, PP, MBP: systolic, diastolic, pulse and mean pressure (p: peripheral, c: central). AIx, AIx@75: augmentation index without and with heart rate adjustment. AP: augmented pressure. Pf, Pb: forward and backward pressure components. CCA, CFA: common carotid and femoral artery. EM: elastic modulus. IMT: intima-media thickness. cfPWV: carotid-femoral pulse wave velocity. DD, SD: diastolic and systolic diameter. *R*: Pearson coefficient. *p <* 0.05 (red) was considered significant. Partial correlations controlling for: ^1^ BW-for-BH (lenght) z-score at birth; ^2^ BW-for-BH z-score at birth and z-BMI at the time of CV study (18 y).

**Table 9:** Multiple regression analysis between CVvariables (dependent) and anthropometric and CRFsvariables (independent) in children cohort (*n* = 632).

Dependent	Independent Variables	βu	SE	βs	*p*	VIF	Adj*R*^2^
Aortic and peripheral blood pressure, hemodynamic and wave reflection parameters							
z-pSBP	Constant	−0.069	0.097		0.477		0.071
	Current z-BMI	0.245	0.062	0.276	**<0.001**	1.00	
z-pDBP	Constant	−0.114	0.108		0.296		0.038
	Current z-BMI	0.203	0.069	0.208	**0.004**	1.00	
z-pPP	-	–	–	–	–	–	–
z-pMBP	Constant	−0.112	0.107		0.295		0.065
	Current z-BMI	0.247	0.068	0.255	**<0.001**	1.00	
z-cSBP	Constant	0.001	0.054		0.985		0.500
	z-pSBP	0.643	0.048	0.707	**<0.001**	1.00	
z-cDBP	Constant	−0.033	0.068		0.625		0.223
	z-pSBP	0.438	0.060	0.477	**<0.001**	1.00	
z-cPP	Constant	0.045	0.071		0.527		0.104
	z-pSBP	0.296	0.063	0.330	**<0.001**	1.00	
z-AIx	Constant	−0.059	0.072		0.414		0.099
	z-pSBP	−0.293	0.064	−0.323	**<0.001**	1.00	
z-AIx@75	Constant	−0.036	0.073		0.627		0.082
	z-pSBP	−0.270	0.066	−0.295	**<0.001**	1.00	
z-AP	Constant	−0.07	0.07		0.336		0.082
	z-pSBP	−0.23	0.06	−0.25	**<0.001**	1.00	
	z-BWH at birth	−0.17	0.07	−0.17	**0.018**	1.00	
z-Pf	Constant	0.069	0.073		0.343		0.149
	z-pSBP	0.361	0.065	0.386	**<0.001**	1.00	
z-Pb	Constant	−0.028	0.077		0.720		0.035
	z-pSBP	0.189	0.069	0.201	**0.007**	1.00	
Structural arterial parameters, local and regional arterial stiffness							
z-R-CCA SD	Constant	−0.222	0.127		**0.084**		0.173
	Current z-BMI	0.335	0.089	0.399	**<0.001**		
	z-pSBP	−0.205	0.091	−0.238	**0.027**		
z-R-CCA DD	Constant	−0.209	0.128		0.107		0.141
	Current z-BMI	0.325	0.090	0.385	**0.001**	1.05	
	z-pSBP	−0.200	0.092	−0.231	**0.033**	1.05	
z-R-CCA IMT	Constant	−0.147	0.146		0.317		0.117
	**ΔBWH z-score (0–2y)**	0.404	0.115	0.513	**0.001**	1.93	
	z-BWH at birth	0.334	0.159	0.306	**0.039**	1.93	
z-L-CCA SD	Constant	−0.121	0.096		0.207		0.100
	Current z-BMI	0.249	0.061	0.326	**<0.001**	1.00	
z-L-CCA DD	Constant	−0.071	0.086		0.414		0.099
	z-BMI	0.339	0.080	0.467	**<0.001**	1.22	
	**Δ** z-BMI (0–6y) * current z-BMI	−0.050	0.023	−0.239	**0.032**	1.22	
z-L-CCA IMT	Constant	−0.118	0.123		0.340		0.029
	z-BMI	0.180	0.078	0.190	**0.024**	1.00	
z-R-CFA SD	Constant	−0.077	0.127		0.545		0.169
	**Δz-BMI (0–6y)**	0.347	0.090	0.411	**<0.001**	1.00	
z-R-CFA DD	**Constant**	−0.086	0.127		0.498		0.157
	**Δz-BMI (0–6y)**	0.347	0.090	0.410	**<0.001**	1.00	
z-R-CFA IMT	**Constant**	0.378	0.242		0.144		0.259
	**Δz-BMI (0–6y)**	−0.751	0.319	−0.562	**0.036**	1.00	
z-L-CFA SD	Constant	−0.161	0.113		0.157		0.034
	Current z-BMI	0.176	0.073	0.202	**0.018**	1.00	
z-L-CFA DD	Constant	−0.138	0.112		0.219		0.027
	Current z-BMI	0.159	0.073	0.185	**0.030**	1.00	
z-L-CFA IMT	Constant	−0.046	0.175		0.796		0.222
	z-pSBP	−0.448	0.147	−0.499	**0.005**	1.00	
z-R-CCA EM	Constant	0.028	0.108		0.794		0.07
	z-pSBP	0.264	0.100	0.29	**0.010**	1.00	
z-L-CCA EM	Constant	0.068	0.085		0.429		0.03
	z-pSBP	0.160	0.075	0.18	**0.034**	1.00	
z-R-CFA EM	-	-	-	-	-	-	-
z-L-CFA EM	-	-	-	-	-	-	-
z-cfPWV	-	-	-	-	-	-	-

βu and βs: un- and standardized coefficients. R: Pearson coefficient. Adj R^2^: adjusted squared R. SE: Standard Error. VIF: variance inflation factor. z-: z-score. BMI: body mass index. SBP, DBP, PP, MBP: systolic, diastolic, pulse and mean pressure (p: peripheral, c: central). AIx, AIx@75: augmentation index without and with heart rate adjustment. AP: augmented pressure. Pf,Pb: forward and backward pressure components. CCA, CFA: common carotid and femoral artery. EM: elastic modulus. IMT: intima-media thickness. DD, SD: diastolic and systolic diameter. cfPWV: carotid-femoral pulse wave velocity. *p <* 0.05 was considered statistically significant. Variables entered in the model (forward method): z-BMI, z-BWH at birth, ΔBWH z-score 0–2y, Δ-zBMI0–6y, Sex (1: female, 0: male), z-pSBP, Hypertension (yes:1, no: 0). Interaction between growth parameters and z-BMI and z-BWH at birth were entered in the model if they showed significant association (*p <* 0.005) in the multiple linear regression. Only significant (*p <* 0.05) variables entered in the model are shown.

**Table 10:** Multiple regression analysis between CVvariables (dependent) and anthropometric and CRFsvariables (independent) in adolescent cohort (*n* = 340).

Dependent Variable	Independent Variables	βu	SE	βs	*p*	VIF	Adj R^2^
Aortic and peripheral blood pressure, hemodynamic and wave reflection parameters							
z-pSBP	Constant	0.259	0.08		**0.001**		0.049
	z-BMI	0.210	0.07	0.209	**0.002**	1.00	
z-pDBP	-	-	-	-	-	-	-
z-pPP	Constant	0.203	0.08		**0.011**		0.025
	z-BMI	0.164	0.07	0.160	**0.017**	1.00	
z-pMBP	Constant	0.201	0.08		**0.010**		0.027
	z-BMI	0.164	0.07	0.165	**0.014**	1.00	
z-cSBP	Constant	0.024	0.05		0.663		0.424
	z-BMI	0.587	0.05	0.653	**<0.001**	1.00	
z-cDBP	Constant	0.039	0.07		0.591		0.178
	z-pSBP	0.422	0.06	0.426	**<0.001**	1.00	
z-cPP	Constant	−0.036	0.06				0.136
	z-pSBP	0.259	0.05	0.313	**<0.001**	1.04	
	z-BMI	0.120	0.05	0.141	**0.030**	1.04	
z-Aix	Constant	0.077	0.07		0.297		0.019
	z-pSBP	−0.126	0.06	−0.138	**0.043**	1.00	
z-Aix@75	-	-	-	-	-	-	-
z-AP	Constant	0.071	0.07		0.324		0.019
	z-pSBP	−0.139	0.06	−0.154	**0.024**	1.00	
z-Pf	Constant	0.015	0.07		0.829		0.098
	z-pSBP	0.270	0.06	0.313	**<0.001**	1.00	
z-Pb	Constant	0.070	0.07		0.334		0.060
	z-pSBP	0.219	0.06	0.245	**<0.001**	1.00	
Structural arterial parameters, local and regional arterial stiffness							
z-R-CCA SD	Constant	0.080	0.08		0.309		0.097
	z-BMI	0.327	0.07	0.311	**<0.001**	1.00	
z-R-CCA DD	Constant	−0.111	0.11		0.313		0.118
	z-BMI	0.325	0.07	0.308	**<0.001**	1.02	
	Sex	0.335	0.15	0.143	**0.029**	1.02	
z-R-CCA IMT	Constant	0.086	0.084		0.308	1.00	0.035
	**Δ** z-BMI 0–6y * current z-BMI	0.134	0.045	0.200	**0.003**		
z-L-CCA SD	Constant	0.052	0.08		0.493	1.02	0.069
	z-BMI	0.255	0.06	0.263	**<0.001**	1.02	
z-L-CCA DD	Constant	−0.113	0.10		0.267		0.101
	z-BMI	0.252	0.06	0.267	**<0.001**		
	Sex	0.298	0.14	0.139	**0.036**	1.00	
z-L-CCA IMT	Constant	0.129	0.09		0.131		0.033
	z-BMI	0.196	0.07	0.182	**0.008**	1.00	
z-R-CFA SD	Constant	0.286	0.11		**0.013**		0.239
	z-BMI	0.343	0.10	0.331	**0.001**	1.03	
	Smoking	−0.609	0.22	−0.275	**0.006**	1.02	
	z-pSBP	−0.222	0.10	−0.223	**0.025**	1.01	
z-R-CFA DD	Constant	0.249	0.12		**0.035**		0.184
	z-BMI	0.294	0.10	0.281	**0.006**	1.03	
	Smoking	−0.669	0.22	−0.299	**0.004**	1.02	
	z-pSBP	−0.201	0.10	−0.200	**0.047**	1.01	
z-R-CFA IMT	Constant	0.643	0.23		**0.011**		0.444
	z-BMI	0.594	0.18	0.559	**0.004**	1.02	
	Sex	–1.067	0.39	−0.453	**0.014**	1.02	
z-L-CFA SD	Constant	0.140	0.12		0.229		0.066
	z-BMI	0.303	0.12	0.278	**0.010**	1.00	
z-L-CFA DD	Constant	0.077	0.12		0.513		0.065
	z-BMI	0.304	0.12	0.275	**0.011**	1.00	
z-L-CFA IMT	Constant	0.175	0.231		0.456		
	Hypertension	5.293	1.083	0.694	**0.000**	1.002	0.557
	Dyslipemia	2.836	1.083	0.372	**0.016**	1.002	
z-R-CCA EM	Constant	−0.061	0.07		0.377		0.099
	z-BMI	0.213	0.06	0.233	**0.001**		
	z-pSBP	0.149	0.06	0.168	**0.012**		
z-L-CCA EM	Constant	−0.267	0.12		**0.030**		0.147
	z-pSBP	0.356	0.11	0.334	**0.002**	1.00	
z-R-CFA EM	Constant	−0.267	0.12		**0.030**		0.111
	z-pSBP	0.356	0.11	0.334	**0.002**	1.00	
z-L-CFA EM	Constant	−0.403	0.11		**0.001**		0.060
	z-pSBP	0.251	0.10	0.267	**0.015**	1.00	
z-cfPWV	Constant	−0.055	0.07		0.401		0.069
	z-pSBP	0.224	0.06	0.263	**<0.001**	1.00	

βu and βs: un- and standardized coefficients. R: Pearson coefficient. Adj R^2^: adjusted squaredR. SE: Standard Error. VIF: variance inflation factor. z-: z-score. BMI: body mass index. SBP, DBP, PP, MBP: systolic, diastolic, pulse and mean pressure (p: peripheral, c: central). AIx, AIx@75: augmentation index without and with heart rate adjustment. AP: augmented pressure. Pf, Pb: forward and backward pressure components. CCA, CFA: common carotid and femoral artery. EM: elastic modulus. IMT: intima-media thickness. DD, SD: diastolic and systolic diameter. cfPWV: carotid-femoral pulse wave velocity. *p <* 0.05 was considered statistically significant. Variables entered in the model (forward method): z-BMI, BWH at birth, Δ-zBMI0–6y, Sex (1: female, 0: male), z-pSBP, Hypertension (1: yes, 0: no), Dislypemia (1: yes, 0: no), Smoking (1: yes, 0: no), Sedentarism (1: yes, 0: no). Interactions between growth parameters and z-BMI or z-BWH were entered in the model if they showed significant association in multiple linear regressions. Only significant (*p <* 0.05) independent variables entered in the models are shown.

## Supplementary Materials

The following are available online at https://www.mdpi.com/2308-3425/6/3/33/s1. Table S1. Clinical, anthropometric hemodynamic, structural and stiffness parameters forchildren and adolescent reference subgroups; Table S2. Hemodynamic, structural and stiffness parameters z-score, forchildren and adolescent Cohorts; Table S3. Multiple linear regression analysis between CVparameters z-scores (dependent variables) and anthropometric parameters (independent variables), children cohort (*n* = 632); Table S4. Multiple linear regression analysis between CV parameters z-scores (dependent variables) and anthropometric parameters (independent variables), children cohort (*n* = 632); Table S5. Multiple linear regression analysis between CV parameters z-scores (dependent variables) and anthropometric parameters (independent variables), children cohort (*n* = 632); Table S6. Multiple linear regression analysis between CV parameters z-scores (dependent variables) and anthropometric parameters (independent variables), children cohort (*n* = 632); Table S7. Multiple linear regression analysis between CV parameters z-scores (dependent variables) and anthropometric parameters (independent variables), adolescent cohort (*n* = 340); Table S8. Multiple linear regression analysis between CV parameters z-scores (dependent variables) and anthropometric parameters (independent variables), adolescent cohort (*n* = 340); Table S9. Multiple linear regression analysis between CV parameters z-scores (dependent variables) and anthropometric parameters (independent variables), adolescent cohort (*n* = 340); Table S10. Multiple linear regression analysis between CV parameters z-scores (dependent variables) and anthropometric parameters (independent variables), adolescent cohort (*n* = 340); Table S11. Multiple linear regression analysis between CV parameters z-scores (dependent variables) and anthropometricparameters (independent variables), adolescent cohort (*n* = 340); Table S12. Multiple linear regression analysis between CV parameters z-scores (dependent variables) and anthropometric parameters (independent variables), adolescent cohort (*n* = 340); Table S13. Multiple linear regression analysis between CV parameters z-scores (dependent variables) and anthropometric parameters and CVRFs (independent variables), children cohort (*n* = 632) (Enter Method); Table S14. Multiple linear regression analysis between CV parameters z-scores (dependent variables) and anthropometric parameters and CVRFs (independent variables), adolescent cohort (*n* = 340) (Enter Method)

## Abbreviations

AIx	central (aortic) augmentation index
AIx@75	AIx adjusted to a 75 beats/min heart rate
AP	central (aortic) augmented pressure
BH	body height
BMI	body mass index
BW	body weight
BWH	body weight for body height
cBP	central (aortic) blood pressure
CCA	common carotid artery
CFA	common femoral artery
cfPWV	carotid-femoral pulse wave velocity
CRFs	cardiovascular risk factors
CV	cardiovascular
CVD	cardiovascular disease
DD	diastolic arterial diameter
EM	pressure-strain arterial elastic modulus
HBP	high blood pressure levels
HR	heart rate
IMT	intima-media thickness
LBW	low birth weight
MLR	multiple linear regression models
mos.	Months
MV	mean value
Pb	amplitude of the cBP backward component
pBP	peripheral (brachial) blood pressure
pDBP	peripheral (brachial) diastolic blood pressure
Pf	amplitude of the cBP forward component
pMBP	peripheral (brachial) mean blood pressure
pPP	peripheral (brachial) pulse pressure
pSBP	peripheral (brachial) systolic blood pressure
PWA	pulse wave analysis
SD	systolic arterial diameter
STD	standard deviation
VIF	variance inflation factor
y	years old
z	z-score
